# Metabolomics for Plant Improvement: Status and Prospects

**DOI:** 10.3389/fpls.2017.01302

**Published:** 2017-08-07

**Authors:** Rakesh Kumar, Abhishek Bohra, Arun K. Pandey, Manish K. Pandey, Anirudh Kumar

**Affiliations:** ^1^Department of Plant Sciences, University of Hyderabad (UoH) Hyderabad, India; ^2^International Crops Research Institute for the Semi-Arid Tropics (ICRISAT) Hyderabad, India; ^3^Crop Improvement Division, Indian Institute of Pulses Research (IIPR) Kanpur, India; ^4^Department of Botany, Indira Gandhi National Tribal University (IGNTU) Amarkantak, India

**Keywords:** biofortification, crop improvement, metabolomics, phytonutrient, fruit quality

## Abstract

Post-genomics era has witnessed the development of cutting-edge technologies that have offered cost-efficient and high-throughput ways for molecular characterization of the function of a cell or organism. Large-scale metabolite profiling assays have allowed researchers to access the global data sets of metabolites and the corresponding metabolic pathways in an unprecedented way. Recent efforts in metabolomics have been directed to improve the quality along with a major focus on yield related traits. Importantly, an integration of metabolomics with other approaches such as quantitative genetics, transcriptomics and genetic modification has established its immense relevance to plant improvement. An effective combination of these modern approaches guides researchers to pinpoint the functional gene(s) and the characterization of massive metabolites, in order to prioritize the candidate genes for downstream analyses and ultimately, offering trait specific markers to improve commercially important traits. This in turn will improve the ability of a plant breeder by allowing him to make more informed decisions. Given this, the present review captures the significant leads gained in the past decade in the field of plant metabolomics accompanied by a brief discussion on the current contribution and the future scope of metabolomics to accelerate plant improvement.

## Introduction

Recent years have witnessed huge developments in different ‘Omics’ fields, namely genomics, transcriptomics, epigenomics, proteomics, metabolomics and phenomics. The information generated by these ‘Omics’ approaches has enhanced precision and speed to the ongoing breeding programs in developing climate smart and nutrition rich germplasm, which is key for ensuring food security (Parry and Hawkesford, 2012). In recent times, the role of phenomics-based breeding has become evident in improving the crop’s performance, and similarly, genomics has made notable contribution in achieving higher genetic gains (Khush, 2001; Langridge and Fleury, 2011; Wang et al., 2017; Xavier et al., 2017). Nevertheless, the diverse omics platforms have great potential in improving the current understanding of important traits, enabling us to develop new strategies for plant improvement. Among omics approaches, the metabolomics is the most complex and has received inadequate attention in crop science, particularly for trait mapping and plant selections.

Metabolites are indispensable component of plant metabolism owing to their influence on plant biomass and architecture (Turner et al., 2016). In recent years, metabolomics has established itself as one of the major breakthroughs in science, paving the way for accurate metabolite profiling in microbes, plants and animals (Heyman and Dubery, 2016; van Dam and Bouwmeester, 2016; Wuolikainen et al., 2016). Metabolomics has the ability to detect a vast array of metabolites from a single extract, thus allowing speedy and precise analysis of metabolites. In other words, metabolomics offers a comprehensive view of cellular metabolites like small organic compounds, which participate in different cellular events, thus representing the absolute physiological state of a cell. In view of the rapidly advancing metabolomics, the metabolite investigation of mutants and transgenic lines holds potential to understand the metabolic networks and to pinpoint the underlying candidate gene(s) (Fernie, 2003; Yonekura-Sakakibara and Saito, 2006; Hong et al., 2016). Also, the metabolomics helps to resolve gene‘s’ function: how a particular gene impacts upon the metabolic pathway, and uncovers different layers of regulation and interception between linked pathways (Wen et al., 2015), which otherwise is difficult to achieve by conventional assays like microarray (Kusano and Saito, 2012). An integrated approach accommodating inferences from genomics, transcriptomics, proteomics, and metabolomics will allow researchers for cataloging and prioritizing the genes to improve important traits in crop species. Further, above-mentioned omics studies have been further extended to explore the associated regulatory steps such as epigenetic regulation, post-transcriptional and post-translation modification. To this end, the interactome network studies aiming to reveal molecular interactions between biomolecules (nucleic acid, proteins, amino acids, carbohydrates, lipids, etc.) deepen our knowledge about the genotype–phenotype relationship (Vidal et al., 2011; Vadivel, 2015).

Metabolomics is being increasingly used in many crop species irrespective of the availability of transgenic system (Oikawa et al., 2008; Fernie and Schauer, 2009; Daygon and Fitzgerald, 2013; Simó et al., 2014). The metabolomics has the potential to facilitate selection of superior traits and improvement of breeding materials (Zivy et al., 2015). In conjunction with the advances in metabolomics, the availability of whole genome sequence, genome-wide genetic variants and cost-effective genotyping assays opens exciting opportunity to effectively integrate metabolomics in crop breeding programs (Hall et al., 2002; Fernie and Schauer, 2009).

The methods and tools employed in metabolomics study, including the mass spectrometry (MS) and nuclear magnetic resonance (NMR) spectroscopy have witnessed substantial improvement. The currently available metabolomics platforms have the capacity to allow large-scale metabolite surveys covering both known as well as unknown metabolites. The deluge of such data, however, makes annotation of metabolites a considerable challenge (Matsuda et al., 2010; Lei et al., 2011). In this context, the ever growing strength of bioinformatics tools coupled with the establishment of metabolomics databases such as the one for model plant Arabidopsis^1^, and others for various plant species^2^ have greater implications for metabolite annotation (Tohge and Fernie, 2009; Afendi et al., 2012). A considerable amount of data have resulted from metabolic surveys, which might support plant improvement schemes focusing on the traits of agricultural importance such as yield and stress tolerance. Further, rapid generation of genome scale data by sequencing of DNA and RNA, and by MS quantification of proteins and metabolites necessitates integration of these information in order to devise a holistic way of improving traits of interests (Pandey et al., 2016). Although, most of the current studies are coming in well-established model organisms, such studies may be of common occurrence in other plant species as well. Scientific community currently faces a herculean challenge of dealing with massive multi-omics data for conducting systems-level analyses (Suravajhala et al., 2016). In such scenario, improved statistical and bioinformatics tools will be required to analyze these data sets together for better consolidation, which can eventually be translated for improving plant performance. In this review, we briefly describe about the latest investigations on plant metabolites and the application of metabolomics including metabolic engineering for plant improvement.

## Analytical Tools for Metabolomic Studies

**FIGURE 1:**
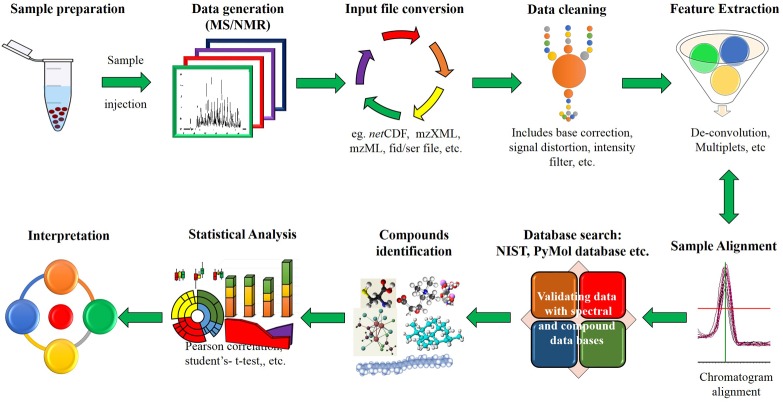
Schematic representation of high throughput data analysis process. A set of raw data files is read after file conversion to desired formats. Data cleaning involves cleaning input file to remove false positives through noise reduction and background correction. Feature extraction is used to differentiate individual peaks from overlapped or closely aligned ones. Additionally, compounds can be identified by analyzing spectra and chemical compound structures available in the metabolomics library or databases.

The modern metabolomics platforms involve generation of metabolome data using two important techniques, namely NMR and MS (**Figure 1**). The NMR based metabolite detection relies upon the utilization of magnetic properties of nuclei of atoms under magnetic field. The NMR is a non-destructive method extensively used to identify metabolites with smaller molecular weight (<50 kDa) for diverse applications like metabolite fingerprinting, profiling, metabolic flux and extracting the atomic structural information of compound present in the biological samples (Winning et al., 2009). However, the poor sensitivity of this technique owing to a limited coverage of low-abundance biomarkers poses a major limitation that in turn restricts its extensive use. Unlike NMR, greater sensitivity of MS allows researchers to attain a wide coverage of metabolome data. This led researchers to identify novel metabolic biomarker, and molecules that can facilitate the reconstruction of metabolic pathways and networks. Recently, MS has achieved greater accuracy with the advances in the ionization methods such as atmospheric pressure chemical ionization (APCI), electrospray ionization (ESI) and MALDI-TOF (Issaq et al., 2009). For enhancing the throughput, MS is usually combined with chromatography techniques such as gas chromatography (GC), liquid chromatography (LC), capillary electrophoresis (CE), fourier transform ion-cyclotron resonance (FT-ICR) and field asymmetric waveform ion mobility spectrometry (FAIMS). Notwithstanding the low sensitivity and large sample requirement of NMR, its capacity of identifying physical properties of ligands, binding sites on protein, uncovering structures of protein ligand complexes and direct binding of target protein retains its use over MS.

The GC-MS platform is widely used for non-targeted analysis (Dutta et al., 2012). GC-MS approach involves derivatization of samples which makes the compounds volatile; however, this leaves underivatized compounds (except hydrocarbon) unnoticed during analysis. Introduction of GC X GC-TOF-MS has notably improved the separation of co-eluting peaks (deconvoluted peak) and also facilitated higher sample throughput (Ralston-Hooper et al., 2008). LC-MS mostly uses ESI and APCI; it has been widely used for targeted and non-targeted approach to detect both primary and secondary metabolites of higher mass i.e., <1500 Da (Turner et al., 2016). Additionally, the combination of UPLC with QTOF-MS has increased the peak resolution, mass accuracy and rapid identification of hundreds of metabolites in a short span of time (Chawla and Ranjan, 2016). In addition to these platforms, CE-MS provides high-resolution separation of different groups of analytes (charged, neutral, polar and hydrophobic) in both targeted and untargeted metabolomics studies (Ramautar and de Jong, 2014). FT-ICR-MS driven by high-resolution mass analysis facilitates detection of large-scale metabolite species with high accuracy (Brown et al., 2005), which could also be combined with separation techniques in order to resolve “very complex matrices” (Schrader and Klein, 2004) and to tackle other issues including ion separation (Lopes et al., 2017). Additionally, FAIMS or differential mobility spectrometry (DMS) an ion mobility based electrophoretic technique coupled with MS. The FAIMS technology was used for the detection of biological samples like volatile compounds formed during bacterial growth (Zrodnikov and Davis, 2012).

Exhaustive data set generated from above high throughput tools are processed through data processing platforms like MET-COFEA, Met-Align, ChromaTOF, MET-XAlign, etc., (Pegasus, 2007; Lommen et al., 2012; Kessler et al., 2014; Zhang et al., 2014, 2015; Ma et al., 2016; Misra and van der Hooft, 2016; de Souza et al., 2017). This basically includes baseline correction, alignment, separation of co-eluting peaks (deconvolution), normalization, etc. (**Figure 1**) prior to identification of compounds. Metabolome databases like METLIN, NIST, GOLM etc., can be used for identification of metabolites (Johnson and Lange, 2015). Further, the identified metabolites data are subject to statistical analysis such as correlation map, principal component analysis (PCA), partial least squares (PLS), K-means clustering, boxplot, heatmap, reconstructing metabolic pathways etc., by using web tool and softwares such as MetaboAnalyst, Cytoscape, Statistical analysis tool etc., (Tsugawa et al., 2015; Xie et al., 2015). These analyses are useful to monitor and identify metabolic markers associated with several agronomic traits.

## Metabolomics for Improvement of Fruits

Metabolomics studies have provided greater insights in fruit biology specially related to ripening and quality. Tomato (*Solanum lycopersicum*) is a rich source of carotenoids, anti-oxidants and flavonoids (Tohge and Fernie, 2015). Metabolite segregation pattern of 50 tomato cultivars showed close agreement with segregation of fruit’s size (Moco et al., 2008). Metabolome is useful to dissect the ripening event by plotting a correlation with fruit transcriptome (Carrari et al., 2006; Osorio et al., 2011). Metabolome can be used to elucidate diverse and differential biochemical pathways exist in the fruits of tomato ILs and Ecotypes (Toubiana et al., 2012; Upadhyaya et al., 2017), and the ancestral species through genome wide metabolic survey (Perez-Fons et al., 2014).

Apple (*Malus* spp.) contains beneficial nutrients in the peel and flesh, including antioxidants that reduce the risk of chronic diseases such as asthma, cancer, cardiovascular disease, and diabetes (Boyer and Liu, 2004). The metabolite contents of the apple fruits are used to differentiate commercially important cultivars (Cuthbertson et al., 2012). For example, the cultivar ‘Golden Delicious’ contains a high load of myo-inositol, sugars and succinic acid; whereas, the cultivars ‘Red Delicious’ and ‘Fuji’ show relatively higher abundance of triterpene/sterols, flavonoids, phenolic acids, stearic acid, anthocyanins, and carbohydrates. The fruit peel extract of ‘Fuji’ contained high levels of carbohydrate including glucose and sorbitol, and was significantly differentiated from ‘Red Delicious’ and ‘Granny Smith’ which contain high levels of unsaturated fatty acids (oleic and linoleic acid). Spatial distribution of sugars and organic acids between fruit layers has been elucidated in a recent study on metabolic profiling of apple fruit (Cebulj et al., 2017). The browning of apple fruits during storage renders them unmarketable, thus exerting an adverse impact on the apple industry. The metabolomics study on stored apple fruits showed a difference in the level of primary metabolites with different time duration (Hatoum et al., 2014). The increased levels of mannose and xylose during post-harvest indicated a breakdown of cell wall hemicellulose, which enhances fruit senescence. The study by Hatoum et al. (2016) established a relation between metabolic regulation during post-harvest storage and cellular respiration and stress.

In recent years, the Kiwifruit (*Actinidia Lindl*. spp.) has gained popularity in international markets due to its distinct appearance and the health benefiting nutrients such as vitamin C and fiber (Ward and Courtney, 2013). A total of 51 metabolites were detected during kiwifruit development and ripening (Nardozza et al., 2013). The content of soluble sugars and ascorbate significantly changes during ripening, which eventually determines the fruit quality and taste (Nardozza et al., 2013). Hence, the quality and flavor of Kiwifruit can be improved by targeting the metabolites that can render consumer acceptance. In Kiwifruit, application of synthetic cytokinin *N*-(2-chloro-4-pyridyl)-*N′*-phenylurea significantly increases fruit size, and affects the ripening processes by altering the accumulation pattern of metabolites such as amino acids, sugars, organic acids etc., (Ainalidou et al., 2015).

The quality and taste of orange (*Citrus* spp.) fruit depend on the composition of metabolites such as organic acids, sugars, vitamins, flavonoids, and carotenoids. The metabolomics study of orange bud mutant ‘Hong Anliu’ (accumulates higher levels of lycopene and sweeter than wild type) led to the identification of 130 metabolites that include acids, sugars, flavonoids, alkaloids, limonoids, coumarins, amino acids, and plant hormones (Liu et al., 2007; Pan et al., 2014). The flavor and the taste of ‘Hong Anliu’ sweet orange was determined by the higher levels of soluble sugars and lower levels of organic acids along with differential levels of flavonoids at ripe stage.

The infection of *Candidatus* Liberibacter asiaticus, causal agent of Citrus Huanglongbing disease, deteriorates juice quality (Slisz et al., 2012). The infection leads to severe decrease in glucose, fructose, sucrose and amino acids such as alanine, arginine, isoleucine, leucine, proline, threonine, and valine; whereas, it enhances the levels of citrate and phenylalanine. Heat treatment of fruit is widely used as a means to avoid fruit infection during post-harvest storage, which is well supported by metabolomics study. In a study, the heat treatment significantly decreased the content of organic acids and amino acid; however, it promoted the accumulation of metabolites such as 2-keto-D-gluconic acid, tetradecanoic acid, oleic acid, ornithine, succinic acid, myo-inositol, glucose, fructose, sucrose, and turanose, which reduces the risk of post-harvest infection (Yun et al., 2013). Recently, ABA is reported to serve as a regulator of citrus cuticular wax biosynthesis during fruit development (Wang et al., 2016).

In grape (*Vitis vinifera*), the fruit setting relies upon the abundance of metabolites, and is regulated by the reprograming of hormones and sugar metabolism pathways (Domingos et al., 2016). The effect of geographical distribution on grape metabolite content is well documented (Son et al., 2009). The grapes grown in the regions perceiving high sun light-low rainfall show enhanced content of sugars and amino acids, Na and Ca, along with low levels of organic acids, suggesting the role of extrinsic factors on grape fruit qualities. The metabolite abundance in grapes berry that is reported to be stage specific and cultivar dependent, regulates the ripening processes (Cuadros-Inostroza et al., 2016). Stilbenes are the major polyphenols present in the grapes that determine the quality of drinking wine. The MS analysis of grapes allowed the detection of several bioactive stilbenes like ampelopsin H, caraphenol, isohopeaphenol, trans-resveratrol, *Z*- and *E*-astringin, piceatanno, *Z*- and *E*-piceid, B pallidol and pallidol-3-*O*-glucoside and parthenocissin A (Flamini et al., 2015). The study focused on the polyphenolics content of the grape identified upto 450 compounds including anthocyanin, glycoside aroma precursors, flavanols and procyanidins, flavones and flavanones, phenolic acids and stilbenes. Particularly, this study allowed identification of several 100 compounds, which were used to build a new database of putative compounds (Grape Metabolomics).

Pear (*Pyrus communis*), a member of Rosaceae, is grown worldwide for its unique ‘melting’ texture. Japan is one of the largest producers of pears. The metabolomics analysis of pear fruit confirmed differential accumulation of ∼250 metabolites during fruit development and ripening (Oikawa et al., 2015). Ripening of pear fruit manifested accumulation of sugars (e.g., sucrose), sulphur-containing amino acids, phytohormone such as ABA and brassinosteroids. This study reported detection of 15 phytohormones including abscisic acid, auxin, brassinosteroids, gibberellins, jasmonic acid and salicylic acid. The blooming stage shows a substantial increase of the metabolites (amino acids and organic acids), which further decreases during fruit development.

Like pears, strawberry (*Fragaria × ananassa*) is rich in secondary metabolites such as flavonoids. The process of gain and loss of strawberry fruit flavors during evolution and domestication was illustrated by Aharoni et al. (2004). The cultivated species of strawberry predominantly contain terpenoids such as monoterpene linalool and the sesquiterpene nerolidol. Whereas, the wild species were rich in the olefinic monoterpenes and myrtenyl acetate. Surprisingly, these were absent in the cultivated species (Aharoni et al., 2004). The untargeted (GC-MS) and targeted (HPLC) based studies of strawberry fruits were employed at seven different stages of fruit development. The metabolic study revealed a shift in the metabolite content during fruit development and ripening. The strawberry ripening involved rise of free amino acid content, with change in sugar content, including substantial changes in other major metabolic pathways such as ester biosynthesis, shikimate, and tricarboxylic acid (Zhang et al., 2011).

The effect of biotic stress and the fungicide (to avoid biotic stress) on strawberry quality was evaluated by quantitative estimation of primary and secondary metabolites accumulated in the infected and non-infected fruits (Mikulic-Petkovsek et al., 2013). The *Colletotrichum nymphaeae* infection induces accumulation of sugars and reduces the organic acid content. The infected fruits displayed altered content of metabolites such as ellagic acid derivatives, flanonols, flavan-3-ols, oligomeric procyanidins and total phenolics. Recent work by Nagpala et al. (2016) revealed an increase in the polyphenol levels in white-fruited species of strawberry as a result of infection from fungal pathogens *viz. Botrytis cinerea* and *Colletotrichum acutatum*.

## Metabolomics for Improvement of Legume Crops

Forage and grain legumes contribute 27% of the world gross primary crop. The grain legumes alone cater 33% of required human dietary protein, contribute to food security and environmental sustainability (Graham and Vance, 2003; Ramalingam et al., 2015). Notwithstanding the extensively investigated model legumes, metabolomics studies in other legumes remain limited. Concerning model legume, investigation of the effect of rhizobial node factor (Nod) in Medicago revealed a decrease in oxylipins (Zhang et al., 2012). In another study, metabolic profiling of salt tolerant *Lotus* species uncovered a series of changes involving metabolic adjustments of shoot constituent for survival (Sanchez et al., 2011).

Stress conditions such as salinity and anoxia result in an accumulation of alanine, and its biosynthesis co-substrates such as glutamate and GABA, and succinate in soybean (Rocha et al., 2010b). Differential expression was also obtained for genes involved in nitrogen fixation and fermentation in root. Interestingly, a negative correlation was observed for the amino acid derived from glycolysis and the TCA cycle during water logging, and several TCA cycle enzymes were induced upon exposure to water logging (Rocha et al., 2010a). Likewise, an attempt to elucidate the metabolic changes associated with flooding stress in soybean led authors to identify a set of 81 mitochondria associated metabolites, thus suggesting a boost in concentrations of metabolites involved in respiration and glycolysis such as, amino acids, NAD and NADH coupled with the depletion of free ATP (Komatsu et al., 2011). Under drought and salinity conditions, metabolite phenotyping of four different Mediterranean accessions of lentil suggested a decrease in intermediates of the TCA cycle and glycolytic pathway (Muscolo et al., 2015). Importantly, this study yielded metabolite markers for specific stress; such as threonate, asparagine/ornithine and alanine/homoserine for NaCl, drought and salinity, respectively. Another study that aimed to assess the impact of water deficiency on *Lupinus albus* demonstrated plant stem serving as a storage organ for sugars and amino acids (Pinheiro et al., 2004). Importantly, tolerant plant accumulated significantly higher level of metabolites such as asparagine, proline, sucrose and glucose in the stem stelar region (Pinheiro et al., 2004). The authors proposed reorganization of nitrogen and carbon metabolism pathways in plants in order to tolerate salinity stress. In soybean, consistent increase in pinitol (sugar alcohol, osmoprotectant) was reported in the tolerant plant at both normal and drought-stressed conditions (Silvente et al., 2012). Similarly, accumulation of sucrose, free amino acids and soluble proteins was observed in tolerant soybean in response to water stress (Tripathi et al., 2015).

## Metabolomics for Improvement of Cereal Crops

Cereals remain the prime source of nutrition worldwide owing to their grains rich in vitamins, minerals, carbohydrates and fats (Sarwar et al., 2013). Cereals have been widely studied in order to quantity variation in metabolites and their association with sequence variation (Chen et al., 2014, 2016). In rice, different research groups have harnessed the potential of metabolomics in order to explore the metabolites diversity between different varieties and natural variants (Kusano et al., 2007; Redestig et al., 2011; Gong et al., 2013; Hu et al., 2014, 2016; Kusano et al., 2015; Chen et al., 2016; Okazaki and Saito, 2016). Similarly, metabolomics studies in maize have allowed researchers to differentiate and subsequently select the superior genotypes with enhanced nutritional composition (Matsuda et al., 2012; Wen et al., 2014; Venkatesh et al., 2016). Recently, metabolomic approach has been utilized to survey chemical diversity between different maize and rice variety and its natural variants (Chen et al., 2016). In maize, drought stress is reported to be regulated by amino acid metabolism (Obata et al., 2015). Photorespiration is tightly regulated under drought as the two amino acids involved in this pathway, glycine and serine are rendered up-regulated. Further, accumulation of glycine and myo-inositol was reported to relate with grain size of maize under drought, implicating these metabolites as potential markers for identifying drought tolerant maize (Obata et al., 2015). Similar work in rice demonstrated drastic induction of certain compounds in tolerant plants such as allantoin, galactaric acid, glucose, gluconic acid, glucopyranoside and salicylic acid, which could be considered as metabolite markers to address drought stress in rice (Degenkolbe et al., 2013). As demonstrated in sorghum by Ogbaga et al. (2016), the plant’s ability to acquire and reorganize its metabolic status in order to cope with drought shows considerable variation within species. Under drought condition, sorghum variety having a greater tolerance to drought (Samsorg 17) accumulated sugars and sugar alcohols in comparison with less drought tolerant variety (Samsorg 40) that accumulated free amino acids. Marked abundance of soluble sugars with amino acids was also observed in the roots of tolerant barley plants under salinity stress (Shelden et al., 2016). Like drought, chilling stress is also known to induce accumulation of amino acids and carbohydrates. For example, chilling stress caused substantial changes in metabolic profiles of rice varieties *viz.* Nipponbare (*Japonica*) and 19-11 (*Indica*) (Zhang et al., 2016). The chilling tolerance of Nipponbare involved metabolic adjustment to activate antioxidation pathway by modulating key metabolites such as γ-glutamylisoleucine, γ-glutamylglutamine, 5-oxoproline, glycine, glutamate, adenine dinucleotide and putrescine (Zhang et al., 2016). Further, chilling stress activates glycolytic pathway, however, normal activity is resumed during recovery phase. In both wheat and barley, cold stress expedites the amino acid pool and induces the GABA-shunt genes to promote conversion of glutamate to GABA (Sutka and Snape, 1989; Mazzucotelli et al., 2006). It is well established that cereal grains accumulate flavones/flavone-glycosides, which protects plants from various stresses (Caasi-Lit et al., 2007). For example, rice produces plenty of flavone-glycosides to protect it from abiotic stress and herbivores (Adjei-Afriyie et al., 2000; Matsuda et al., 2012). However, examination of herbivore-induced defense system in maize showed an increase in azealic acid, *N*-hydroxycinnamoyl tyramines, phospholipids, tryptophan, and 1,3-benzoxazin-4-ones (Marti et al., 2013). Accumulation of resistance related metabolites is also reported during plant–pathogen interaction. For instance, a tolerant variety of wheat can accumulate a wide range of metabolites conferring tolerance such as coumaroylputrescine and coumaroylagmatine during fusarium head blight (Kage et al., 2016). Further, evaluation of these hydroxycinnamic acid amide compounds and their placement on metabolic pathways has led to the identification of an important gene *agmatine coumaroyl transferase* (*ACT*).

## Impact of High CO_2_ Stress on the Metabolome and its Attributes Toward Quality and Yield

According to a report of the intergovernmental panel on climate change (IPCC), anthropogenic activity, deforestation and combustion of fossil fuel could boost CO_2_ level upto 700 ppm by 2100 (IPCC, 2013)^3^. The CO_2_ uptake and water availability are directly connected to photosynthesis and plant growth, and CO_2_ sequestration by plants helps in maintaining terrestrial ecosystems (Weltzin et al., 2003; Reich et al., 2006; Arora and Boer, 2014). A recent study by Liu et al. (2016) has shown the impacts of elevated atmospheric CO_2_ on plant growth rate, biomass and leaf area. Terrestrial plants and phytoplankton significantly utilize increased atmospheric CO_2_ to increase their biomass (Lawlor and Mitchell, 1991; Schippers et al., 2004; Forkel et al., 2016). However, enhanced CO_2_ levels might promote grass species in the long term (Smith et al., 2000), which is an encouraging finding concerning food crops such as cereal. Sustaining crop performance in the face of growing CO_2_ levels remains a key challenge of 21st century agriculture. Therefore, studies are required to understand the metabolic composition and the relevant alterations on metabolome due to high CO_2_ stress.

### Effect on Quantity

Fruit, grain and tuber are the ultimate sink organs of the plant. The growth of these sink organs is directly depends on the partitioning of photosynthate from source organ to sink (Marcelis, 1996; Osorio et al., 2014). The sink organs store variety of metabolites which depends on species, source strength, composition of allocated photosynthate and plant requirement (Edson et al., 1995; Heuvelink, 1997; Cuzzuol et al., 2005; Kanai et al., 2007; Albacete et al., 2014; Li et al., 2015). To date several reports have been published that have focused on the correlation of high CO_2_ with yield (harvesting sink organ) in commercial crop species (Schippers et al., 2004). For instance, high CO_2_ was reported to cause a significant increase in productivity due to the increased level of photosynthesis in rice, wheat and soybean (Teramura et al., 1990). More recent studies in wheat and rice validated stimulation of yield under greater amount of atmospheric CO_2_ (Cai et al., 2016; Fitzgerald et al., 2016). Though, the increase in soybean was quite consistent, it was not as significant and high as reported in the case of rice and wheat (Morgan et al., 2005; Ziska and Bunce, 2007). Coupling enrichment of CO_2_ with drought stress in barley to examine yield loss rescue ability of elevated CO_2_ suggested that modern barley cultivar could perform better under climate change (Schmid et al., 2016). Similar result was obtained earlier in potato in which enriched CO_2_ farming led a 54% increase of tuber yield (Miglietta et al., 1998). Likewise, enhanced CO_2_ level registered higher yield in cotton, however, it was lower than the yield obtained under elevated temperature (Osanai et al., 2017).

### Effect on Quality

Obtaining crop produce with high quality also remains a global concern, especially at a time when a substantial proportion of the population worldwide is affected with nutrition related disorders (Bohra and Singh, 2015). Though enhanced yield was witnessed as a result of elevated CO_2_, will this be able to meet the demand concerning nutritional quality and food security as most of these studies are being conducted in cereals that are rich in carbohydrate. Also, though elevated CO_2_ in atmosphere increases yield, it affects the C/N ration in C_3_ and C_4_ plants by altering nitrate assimilation (Taub and Wang, 2008; Bloom et al., 2012). For instance, as shown by Bloom et al. (2014), wheat grown in the elevated CO_2_ open field condition manifests slower nitrate metabolism. The reduced nitrogen in cereal results from increased levels of carbohydrates (Idso and Idso, 2001). Metabolomics studies of wheat grown under CO_2_ enriched atmosphere have shown a substandard accumulation of amino acids, and a significant increase in fructose, fructan and lipidic content in grains (Högy et al., 2009, 2010b). In soybean leaves, ureide (derived from urea) and total amino acid levels were increased at the early season, but, later it resumed to initial level (Rogers et al., 2006). Similarly, a combination of temperature and elevated CO_2_ efficiently decreases the levels of amino acids in root of Chinese cabbage (Reich et al., 2016). In the strawberry fruit, elevated CO_2_ and high temperature increase the sugar and sweetness index along with a reduction in the antioxidant and nitrogen content (Sun et al., 2012b). CO_2_ enrichment has also shown encouraging results in other crops, such as increase of vitamin A and C in tomato, and vitamin C in orange fruit (Idso and Idso, 2001). Taken together, it becomes evident that crop grown in elevated CO_2_ obtains higher yield to a certain extent; however, this may drastically affect the nutritional content, especially nitrogenous amino acids.

Elevated CO_2_ was reported to exert a huge impact on mustard seed oil quality due to an increase in starch and oil content of seed at the expense of protein. The excess of carbohydrate affects the lipid composition of mustard seed, thus causing an increase in the concentration of oleic acid, and a simultaneous decrease in the content of linolenic acid and nervonic acid (Högy et al., 2010a). The CO_2_ enrichment is reported to reduce the erucic acid (undesired factor) while improving mustard seed quality (Uprety et al., 2010). In groundnut, elevated CO_2_ directed storage of high-quality oil in seeds of two varieties JL 24 and ICGV 91114 (Yadav et al., 2011) corroborated with the results reported in mustard. Similarly, sunflower seed showed a decrease in amino acids, proteins and minerals at high CO_2_ concentration, however, oil load with health-benefiting unsaturated acids was increased (Pal et al., 2014).

## Effect of Biotic and Abiotic Stress on Plant Lipidome

Lipids are an important constituent of cell membrane enclosing organelles and suborganelles, in which various biochemical reactions occur. Modern lipidomics has facilitated profiling of lipids to understand lipid dynamics and biosynthesis on exposure to a range of stresses (Kosma et al., 2010; Burgos et al., 2011; Szymanski et al., 2014; Hou et al., 2016; Li et al., 2016; Tenenboim et al., 2016).

Plants adjust their lipid structure according to varying environmental conditions (Tenenboim et al., 2016). For instance, cold tolerant plants increase the levels of desaturated glycerolipids to maintenance membrane fluidity (Sakamoto et al., 2004; De Palma et al., 2008; Degenkolbe et al., 2012). Freezing plants up to a sublethal temperature can induce the level of lysophospholipids, phosphatidic acid, and phosphatidylglycerol (Welti et al., 2002; Zhang et al., 2013a). In contrast, tolerance to heat stress involves an increase in saturated glycerolipids (Larkindale and Huang, 2004). Recently, MS based analysis revealed remodeling of lipids, antioxidants and galactolipids in tomato plant during high temperature stress (Spicher et al., 2016). A combined glycerolipidomic and transcriptomics study provided insight on lipid remodeling, and regulatory genes involved in lipid biosynthesis and heat stress management (Burgos et al., 2011; Szymanski et al., 2014; Higashi et al., 2015; Légeret et al., 2016; Narayanan et al., 2016a,b). Most strikingly, high temperature can induce dramatic increase of lipid antioxidant such as α-tocopherol and plastoquinone/-ol, and saturation of membrane lipids like galactolipids and phosphatidyl ethanolamine (Spicher et al., 2016).

Hypoxia represents another important type of abiotic stress that plant faces due to excessive watering or flood and leads to limited O_2_ availability and increased salinity for a submerged plant (Rajapakse et al., 2009). According to Xie et al. (2015), plant cell synthesizes and accumulates unsaturated ceramides, a class of sphingolipids under hypoxia. More recently, root lipidic content of two barley genotypes was examined to understand the mechanism underlying their tolerance to salinity stress (Natera et al., 2016). Phosphorus availability during stress is known to directly affect the membrane lipid texture. For instance, Arabidopsis grown in phosphorus-deficient condition can induce replacement of phospholipids with non-phosphorus SQDG class lipids (galactolipids) (Essigmann et al., 1998; Härtel et al., 2000). These galactolipids are mostly associated with plastic thylakoid membrane, and phosphorus deprivation causes its enrichment in the roots extraplastidic membrane for survival.

Compared to abiotic stress, lipidomics studies of biotic stress are scanty. During biotic stress, lipid peroxidation occurs due to formation of reactive oxygen species (ROS), ultimately leading to program cell death (PCD) (Zoeller et al., 2012). A recent report suggests *inositol phosphorylceramide synthase* as instrumental to coordinate the PCD, a mechanism acquired by plants for its self-defense to limit biotrophic pathogens (Wang et al., 2008). Lipid peroxidation results in formation of jasmonate and oxylipins, which are signaling molecules during plant immune response (Shah, 2005). Recently, nearly 100 membrane-associated lipids were quantified in response to methyljasmonate and cerium, suggesting increase of lysophosphatidylcholine, phosphatidic acid and phosphatidylcholine associated with PCD (Yang et al., 2008). Plant–pathogen interaction also impacts upon plant cuticle that serves as a first physical barrier limiting pathogen invasion along with protecting plants from other physical damages (Jenks et al., 1994). The cuticle layered over epidermal cells, is mainly composed of wax and cutin (Samuels et al., 2008; Mazurek et al., 2017). The cutin is mainly composed of hydroxylated C_16_ and C_18_ (Cheng and Walden, 2005; Kunst and Samuels, 2009). The permeability of cuticle relies upon its composition, which can restrict the invasion of fungal pathogens such as *Botrytis cinerea* (Saladié et al., 2007; Curvers et al., 2010). The role of cuticle in relation to plant defense against pathogen invasion is well described in the recent articles (Chassot and Métraux, 2005; Bessire et al., 2007; Chassot et al., 2008; Raffaele et al., 2009; Reina-Pinto and Yephremov, 2009; L’Haridon et al., 2011; Buxdorf et al., 2014; Serrano et al., 2014; Lara et al., 2015; Fernández et al., 2016).

## Integrating Layers of Metabolomics and Other Omics Science

### Epigenetic Modifications and Plant Metabolites

Epigenetic modification refers to DNA methylation and histone modification, which in turn alters the gene expression in a heritable fashion without causing any change in the underlying DNA sequence (Bird, 2007). For example, deformity of the flower in the toadflax (*Linaria vulgaris*) mutant was created due to extensive methylation and suppression of *cyc*-like gene that controls flower symmetry (Cubas et al., 1999). Research during the last decade has led to a significant gain in the knowledge related to epigenetic influence on metabolism, however, most of the studies were confined to animal system (Kaelin and McKnight, 2013; Petersen et al., 2014; Paul et al., 2015). The reason may be less availability of epigenetic mutants in the plants. In plant breeding, the epialleles can serve as a novel source of trait variation.

In Arabidopsis, disruption of *MSH1* caused altered plant growth phenotypes due to hypermethylation of chromosome segments (Virdi et al., 2015). Hypomethylation of *RAV6* promoter in rice *Epi-rav6* mutant resulted altered leaf size and grain size, via modulating brassinosteroid (BR) homeostasis (Xianwei et al., 2015). In maize, the *IPA* mutation affects the biosynthesis and accumulation of phytic acid in the seed, which influences germination along with affecting plant growth and responses to various environmental conditions (Pilu et al., 2009). The maize epigenetic mutation *lpa1-241* leads to drastic reduction of phytic acid and higher level of free inorganic phosphate in seeds. Similarly, epigenetic regulation of maize *booster1*, *Pericarp color1, purple plant1* and *red1* genes impacting upon anthocyanin and flavonoid biosynthesis is well documented (Della Vedova, 2004; Chandler and Alleman, 2008; Mach, 2012). In tomato, whole genome bisulphite sequencing of fruit revealed methylation of 1% of the total genomic region (Zhong et al., 2013). Further, it was demonstrated that epigenetic modification is not static during tomato fruit development and ripening, instead methylation of the promoter region significantly decreases for ripening specific genes such as *ripening inhibitor* (*RIN*) and *colorless non-ripening* (*CNR)*. In fact, DNA methylation regulates fruit phenotype by altering wide range of primary and secondary metabolites in tomato. For example, methylation of SBP-box promoter of epigenetic mutant *Cnr* results in severe decline of ethylene and carotenoids, thus affecting fruit shelf life (Manning et al., 2006). Additionally, the interaction of CNR with RIN affects the expression of ripening related gene (Oa et al., 2011). The *rin* mutant exhibits reduced levels of carotenoids, downregulation of ethylene, amino acids, organic acids and sugars (Osorio et al., 2011). A recent study in tomato has demonstrated that methylation in the promoter region of the gene *2-methyl-6-phytylquinol methyltransferase* (*VTE3*) affects biosynthesis and accumulation of γ- and α-tocopherols (Quadrana et al., 2014). *VTE3* underlies VTE quantitative trait locus (QTL) that is responsible for the modulation of important metabolic QTL. Domestication of allotetraploid cotton has resulted 12 million differentially methylated cytosines, which includes more than 500 genes contributing to agronomyic traits including seed dormancy and flowering time (Song et al., 2017).

### Correlation Analyses of Transcriptomics and Metabolomics

Researchers increasingly focus on correlating metabolome with genomic segments to discover genetic determinants of regulatory pathways to improve compositional quality of crops species (Riedelsheimer et al., 2012; Gong et al., 2013; Strauch et al., 2015). Metabolome study in Arabidopsis has enriched the understanding of the metabolism and biosynthesis of glucosinolate, oil biosynthesis and oligosaccharides in seed (Bentsink et al., 2000; Kliebenstein et al., 2001; Hobbs et al., 2004). Recent advances have revealed an association of genetic variants with metabolites that could be used for metabolic engineering across various plant species such as Arabidopsis, broccoli, maize, mustard, potato, rice, sesame, tomato, and wheat (Schauer et al., 2005, 2006; Kusano et al., 2007; Yonekura-Sakakibara et al., 2007; Laurentin et al., 2008; Rochfort et al., 2008; Tohge and Fernie, 2012; Khakimov et al., 2014; Cho et al., 2016; Wen et al., 2016). For example, study of Arabidopsis ecotypes *Landsberg erecta* from Cape Verdi Islands revealed a strong correlation of *fatty acid desaturase 3* with the unsaturated fatty acid content (linoleic and linolenic acids) in seeds (Hobbs et al., 2004). The flavonoid biosynthesis in Arabidopsis is regulated by gene *flavonol 7-O-rhamnosyltransferase*, its transcripts accumulate with the flavonoid abundance in floral buds (Yonekura-Sakakibara et al., 2007). Another study revealed induction of eight novel anthocyanins out of 1800 metabolites in an overexpression line of MYB transcription factor encoding *PAP1* gene (Tohge et al., 2005). These approaches in tomato and populus enabled gathering in-depth knowledge about the flavonoid biosynthetic pathway (Spencer et al., 2005; Morreel et al., 2006). Concerning the flavonoid metabolism pathway, a strong correlation between transcripts and metabolites was inferred in potato through combining transcriptomics and metabolomics approaches (Cho et al., 2016). The study captured interaction between 22 metabolites and 119 transcripts, which strongly regulate the anthocyanin content of light-red Hongyoung and dark-purple Jayoung potatoes. Analysis of 210 recombinant inbred lines (RILs) derived from Bay × Sha facilitated detection of more than 400 QTLs for 243 metabolites. Total 11 QTL clusters were obtained, of which five overlapped with expression QTLs reported in earlier studies. Importantly, epistatic interactions were noted in eight QTL clusters (Rowe et al., 2008). In a similar fashion, genetic interactions explained the metabolic variation in maize (Wen et al., 2016). Two RIL populations (B73/By804 and Zong3/Yu87-1) were phenotyped for 155 metabolites and detected > 800 QTLs from both populations, majority of which had smaller effect sizes. This work provided deeper insights on flavonoid pathway, highlighting the significance of the *p* locus. Notably, 32 QTLs were cross validated between the two populations, whereas 57 associations detected in the genomic regions that overlapped with the QTLs detected earlier in genome-wide association studies (GWAS) performed by Wen et al. (2014). In rice, 2,800 metabolite QTLs (mQTLs) were detected for 900 metabolites showing strong association with 24 candidate genes involved in various metabolic biosynthetic pathways, including *O*-glycosyl flavonols (Gong et al., 2013). Recently, metabolic profiling of leaf and fruits of five wild relatives of tomato (S. *chmielewskii, S. habrochaites, S. neorickii, S. pennellii*, and *S. pimpinellifolium*) showed a wide range of metabolome variability that are important for stress response, and also contribute to nutritional richness (Schauer et al., 2005). Another study that combined transcriptomics and metabolomics of tomato fruits revealed a strong correlation between the ripening-induced transcripts and metabolites specific to the Krebs cycle and sugars (Carrari et al., 2006). A more recent study consolidating profiling patterns of transcripts, proteins, and metabolites of ripening defective mutants *non-ripening* (*NOR*), *never-ripe* (*Nr*) and *ripening-inhibitor* (*RIN*) suggested shifts in the primary metabolites that eventually reduced metabolic activities during ripening (Osorio et al., 2011). Metabolite QTLs analysis based on metabolic profiling of 76 introgression lines (ILs) of tomato has uncovered a strong regulation of seed metabolism during fruit development (Toubiana et al., 2012). Recently, a tomato Eco-TILLING population showed wide variation in the folate content in the fruits (Upadhyaya et al., 2017). Additionally, the genome-wide metabolomic survey of ILs and the ancestral species *Solanum pennellii* led to identification of important compounds such as Vit-E etc. and importantly, this analysis assigned nearly 2,000 compounds to the tomato genome (Perez-Fons et al., 2014). Interestingly, the segments introgressed from *S. pennellii* (Chromosomes 3, 6, 8, and 12) into ILs were reported to alter isoprenoids and tocopherols content at the fruit level and the study led authors to associate the differential expression of metabolites with photosynthesis and photorespiration. Similarly, metabolic profiling of aneuploid wheat highlighted the genes regulating variation in the branched chain amino acids and accumulation of trehalose in mature grain (Francki et al., 2015).

Further, the use of metabolic information in genome wide predictions, as demonstrated by Riedelsheimer et al. (2012) in hybrid maize, opens up novel opportunities to considerably enhance genetic gains. Similarly, modern techniques such as the epigenome wide association studies (EWAS) employed recently in human (Petersen et al., 2014) may also be extended to plants to better capitalize on the potential of trait-associations like “methylome-metabotype association” for accelerating plant improvement. Additionally, recent interactome network studies, which focus on molecular interactions between biomolecules (nucleic acid, proteins, amino acids, carbohydrates, lipids, etc.) provide deeper insights on correlation between genotype and phenotype (Vidal et al., 2011; Vadivel, 2015; Zivy et al., 2015).

### Combining Proteome Analysis with Metabolite Profiling

**FIGURE 2:**
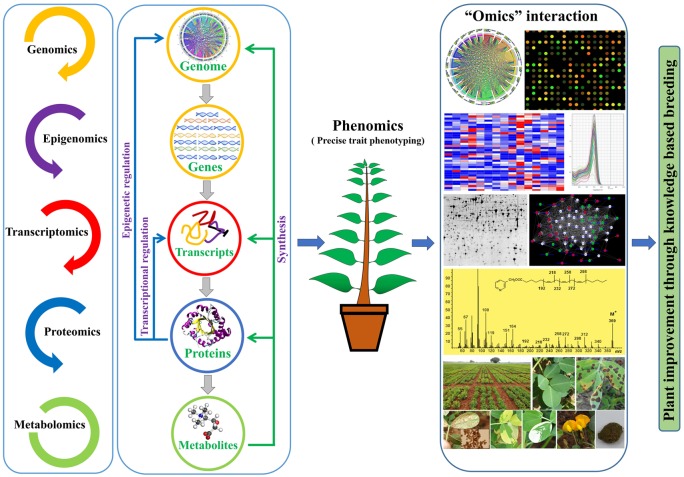
An overview for the use of ‘omics’ approaches for crop improvement.

In addition to genomics, proteomics in combination with other modern high throughput approaches such as genomics and transcriptomics has contributed to revolutionize the omics era, and has paved the way to decipher the complex molecular mechanism underlying various commercial traits (Weckwerth, 2008; Barros et al., 2010; Ricroch et al., 2011; Ramalingam et al., 2015). For instance, the effect of pollutant ozone (O_3_) was studied in rice because it damages cellular tissue by creating ROS, thus altering photosynthetic ability that severely reflects in yield loss. It was reported that exposure of O_3_ to rice leaves significantly induced oligopeptidase-B and proteasome subunit alpha type1, which are involved in the 20S proteasome alpha subunit that mediate ATP dependent protein degradation (Cho et al., 2008). Further, O_3_ exposure induced accumulation of stress and metabolism related proteins such as glutathione peroxidase, aconitate hydratase, fumarylacetoacetase hydrolase, dehydrogenase P protein, and thiamine biosynthetic protein. These protein modulations in O_3_ exposed leaves were concomitant with dramatically increased levels of free amino acids, nucleotides and glutathione. A combined proteomics and metabolomics approach in response to temperature induced stress in Arabidopsis revealed several important markers (Wienkoop et al., 2008). Cold or heat stress induces production of osmolytes (metabolic markers) proline, glutamine, raffinose and galacinol. In Arabidopsis, these metabolites were identified along with protein markers chloroplastidic glyceraldehyde-3-phosphate dehydrogenase (GAPDH), cytosolic GAPDH, chloroplast chaperonin, cyclophilins, protein 78, COR6.6 and several RNA binding proteins. Heavy metals are potential threat to crop productivity, accumulation of these elements causes developmental and physiological changes. For example, cadmium (Cd) accumulation retards plant growth and causes chlorosis (Prasad, 1995; Moulis, 2010). Such investigation has involved several plant species such as Arabidopsis, mustard, soybean, flax, Medicago, rice, pea, tomato, and spinach to understand the cellular responses to Cd stress (Villiers et al., 2011). Cadmium induced toxicity in rice drastically affects the expression of RuBisCO, Calvin’s cycle and kreb’s cycle enzymes, which leads to attenuation of carbohydrate and amino acid metabolism. Further, the use of these platforms has been extended to understand biotic stress (Salekdeh and Komatsu, 2007; Lodha et al., 2013). Study of chickpea roots infected by *Fusarium oxysporum* suggested efficient and increased carbon and nitrogen metabolism, accumulation of phytoalexins, and lignification coupled with enhanced accumulation of proteins related to pathogenesis (Kumar et al., 2016). Similarly, a system biology approach to understand the response of microbial symbioses on the pea plant metabolism under *Didymella pinodes* infection reports systemic resistance via adjustment of proteome and metabolome (Desalegn et al., 2016). The rhizobia associated resistant plants showed induction of amino acid, TCA, and secondary metabolism, including the pisatin, and proteins associated with pisatin biosynthesis. Coupling metabolomics with other omics tool has enabled researchers to acquire deeper knowledge of molecular events involved in important biological process required for plant sustainability (**Figure 2**). As a result, metabolomics has been exploited in several plant species to better understand the biological phenomena including plant development and stress response (**Table 1**).

**Table 1:** List of plant species selected for metabolomics study.

Type of work	Plant name		Type of work	Citation
Fruit metabolome	*Solanum lycopersicum*		Carotenoid study	Long et al., 2006
			Primary metabolite analysis	Moco et al., 2008
			Metabolite QTL mapping	Perez-Fons et al., 2014
	*Malus* spp.		Cultivars differentation	Cuthbertson et al., 2012
			Post-harvest associated metabolic changes	Hatoum et al., 2014
			Metabolic distribution inside the fruit	Cebulj et al., 2017
	*Actinidia Lindl. spp.*		Fruit ripening and development	Nardozza et al., 2013
			Effect of cytokinin on fruit	Ainalidou et al., 2015
	Citrus		Mutant study	Pan et al., 2014
			Effect of GABA on fruit ripening	Sheng et al., 2017
	*Vitis* spp.		Effect of abiotic factor on grape quality	Son et al., 2009
			Identification of stilbenes	Flamini et al., 2015
	*Pyrus communis*		Fruit developmentt and ripening	Oikawa et al., 2015
	*Fragaria spp.*		Domestication and fruit quality	Aharoni et al., 2004
			Fruit development and ripening	Zhang et al., 2011
	*Melon*		Effect of environmental factor on melon quality	Bernillon et al., 2013
	*Capsicum annuum*		Fruit development and ripening	Jang et al., 2015
Stress Management	Biotic stress	*Fragaria spp.*	Effect of Colletotrichum nymphaeae infection	Mikulic-Petkovsek et al., 2013
			Effect of *Botrytis cinerea* and *Colletotrichum acutatum*	Nagpala et al., 2016
		Citrus	Post-harvest infection management	Yun et al., 2013
	Abiotic stress	*Lotus* spp.	Salt tolerance	Sanchez et al., 2011
		Soybean	Flooding stress	Komatsu et al., 2011
			Drought stress	Silvente et al., 2012; Tripathi et al., 2016
		Lentil	Drought and salinity	Muscolo et al., 2015
		*Lupinus albus*	Drought stress	Pinheiro et al., 2004
		Maize	Drought stress	Obata et al., 2015
		Rice	Drought stress	Degenkolbe et al., 2013
			Chilling stress	Zhang et al., 2016
			Herbivores stress	
		Sorghum	Drought stress	Ogbaga et al., 2016
		Barley	Salt tolerance	Shelden et al., 2016
			Chilling stress	Mazzucotelli et al., 2006
		Maize	Herbivores	Marti et al., 2013
		Rice	Herbivores	Adjei-Afriyie et al., 2000
		Rice	Effect of elevated CO_2_ on yield	Cai et al., 2016
		Wheat		Cai et al., 2016
		Barley		Schmid et al., 2016
		Soybean		Morgan et al., 2005
				Ziska and Bunce, 2007
		Potato		Miglietta et al., 1998
		Wheat	Effect of elevated CO_2_ on grain quality	Bloom et al., 2014
		Soybean	Effect of elevated CO_2_ on leaves metabolite	Rogers et al., 2006
		Groundnut	Effect of elevated CO_2_ on seed content	Yadav et al., 2011
		Chinese cabbage	Elevated CO_2_ on cabbage quality	Reich et al., 2016
		Strawberry	Elevated CO_2_ on fruit quality	Sun et al., 2012b
		Mustard	Elevated CO_2_ on seed oil	Chakraborty and Uprety, 2012
Study of trangenic	Oil quality	Jatropha	Lipid analysis	Qu et al., 2012
			Oil quality under drought stress	Tsuchimoto et al., 2012
		Castor	Oil quality and yield under against feeders	Malathi et al., 2006
		Sugarcane	Sugar quality	Arruda, 2012
				Hamerli and Birch, 2011
				Wu and Birch, 2007
		Barley	Sugar metabolism	Willis et al., 2016
		Potato		
		Rice		
		Sunflower		
		Tobacco		
		Arabidopsis	Altering the photosynthate load through *hexokinase1*	Menu et al., 2004
		Tomato	Alteration of sugar and organic acid levels	Nunes-Nesi et al., 2005
		Tomato	Alteration of cell wall non-cellulosic components	Gilbert et al., 2009
	Flavonoid biosynthesis	Apple	Anthocyanin content	Espley et al., 2007
		Tobacco		Takos et al., 2006
		Arabidopsis		Lin-Wang et al., 2010
		Strawberry		Lin-Wang et al., 2014
		Grapes		Walker et al., 2007
		Tomato		Butelli et al., 2008
	Altering the fruit size by targeting multiple genes	Tomato	Altering the fruit load	Baldet et al., 2006
	Fruit shelf life	Tomato	Ethylene metabolism	Picton et al., 1993
		Apple		Wang et al., 2009
	Hormone alteration	Tomato	Fruit size	De Jong et al., 2011
		Strawberry	Flavonoid content	Jia et al., 2011
		Tomato	Drought tolerance	Thompson et al., 2007
		Rice		Xiong et al., 2001
		Canola		Wang et al., 2009
		Barley		Zalewski et al., 2010
		Maize	Flower modification	Makarevitch et al., 2012
		Rice	Senescence	Kang et al., 2009
	Vitamin enrichment	Potato	Vit-A	Diretto et al., 2010
		Rice		Paine et al., 2005
		Lettuce	Folate	Nunes et al., 2009
		Rice		Dong et al., 2014
	Aroma	Tomato	*S*-linalool accumulation	Butelli et al., 2008
		Cucumber	(E, Z)-2,6-nonadienal accumulation	Zawirska-Wojtasiak et al., 2009
		Tomato	Defense mechanism	Domínguez et al., 2010


## Examples of Plant Improvement Through Metabolic Engineering

Metabolites are the ultimate downstream factors that regulate and decide the cell fate; hence the content of metabolite directly affects organ physiology and often signifies the quality of fruits. The improvement of gene annotation is important to validate the gene function. In fact, it facilitates the use of these genes in the field of crop sciences to improve the quality and yield. This section describes significant leads achieved in the field of crop species through metabolic engineering.

### Altering Photosynthate Levels to Change Fruit Dimensions

Fruit development and weight are significantly correlated with the metabolic composition of fruit (Osorio et al., 2014). The development of fruit represents substantial change of organic acid (predominantly citrate and malate) and sugars that determines the final quality of the ripe fruits (Azzi et al., 2015). Unlike leaves, fruits act as a sink and its development depends on the translocation of photo-assimilates of leaves than that of own photosynthesis products (Bénard et al., 2015). The impact of phloem translocate on fruit development and size was evident by concomitant increased growth for both flower and fruits with increased levels of photo-assimilate by reducing the number of flowers or fruits per truss (Baldet et al., 2006). For example, the incubation of tomato plant in the dark significantly reduces fruit size and shape due to repression of cell cycle genes of fruit which severely affects the cell number and cell size (Bohner and Ban, 1988; Bertin et al., 2002; Baldet et al., 2006).

In order to investigate the correlation between sugar content and fruit size, the *hexokinase 1* (*AtHXK1*) of Arabidopsis was over expressed in tomato plant (Menu et al., 2004). The overexpression line showed reduced fruit size due to reduction of cell expansion concomitant with reduced photosynthate. Additionally, transgenic fruits exhibited reduced respiratory rates accompanied by reducing ATP levels. The load of sucrose known to involve in the early stage of fruit development, import of sucrose in fruits is much needed in young fruits that influence fruit set and development (D’Aoust et al., 1999). The establishment of relations between glycolysis, sucrose metabolism and organic acid biosynthesis was profound from the transgenic plants expressing malate dehydrogenase (*mMDH*) in tomato (Nunes-Nesi et al., 2005). The enhanced fruit dry mass of RNAi-*mMDH* plants was concomitant with enhanced photosynthetic ability, which improved carbon assimilation. The silencing of *mMDH* promoted the accumulation of redox stabilizing compounds such as l-galactono-1,4-lactone precursor of ascorbic acid. The silencing of l-galactono-1,4-lactone dehydrogenase (*Gal-LDH*) substantially affecting cell size that resulted in smaller fruits (Alhagdow et al., 2007). Additionally, silencing of the key enzyme of ascorbate biosynthesis GDP-D-mannose 3,5-epimerase (GME) results defect in cell expansion and biosynthesis of cell wall non-cellulosic components (Gilbert et al., 2009). This finding suggests the direct influence of ascorbate in the process of channeling energy during respiration and photosynthesis, and fruit metabolite levels during tomato fruit development (Azzi et al., 2015).

### Exploring *MYB* Transcription Factors to Improve Fruit Quality

In eukaryotes, MYB family transcription factors represent huge family, which controls diverse function such as development, metabolism, and stress related response. Sequencing of the Arabidopsis genome leads to the discovery of the several MYB transcription factors which are mostly characterized to R2R3-MYB family (Dubos et al., 2010). In fruit anthocyanin biosynthesis regulated by R2R3-MYB transcription factor family. The red color of apple skin requires accumulation of anthocyanin, which is controlled by the expression of anthocyanin biosynthetic gene expression. MYB transcription factor *MdMYBA* and *MdMYB10* positively regulates anthocyanin content in apple fruits by binding to the promoter region of anthocyanin biosynthesis genes (Ban et al., 2007; Espley et al., 2007). Interestingly, low temperature and UV-B exposure, enhance the expression of MYB, which enhances anthocyanin accumulation (Ban et al., 2007). Expression of *MdMYBA* under 35S promoter in tobacco results remarkable increase in the anthocyanin content of flowers. MdMYB10 share homology to PAP protein and overexpression of MdMYB10 in apple up-regulate anthocyanin in the whole part of regenerated transgenic plants, including the transformed callus (Espley et al., 2007). In Arabidopsis overexpression of *MdMYB* results elevated level of anthocyanin only in seeds, but not in the leaves. Like tobacco, Arabidopsis also lacks bHLH which interact with MYB to enhance the anthocyanin accumulation (Takos et al., 2006). In addition to bHLH, MYB also interacts with WD-repeat proteins and regulates anthocyanin biosynthesis through “MBW” complex, which is formed of MYB, basic helix-loop-helix (bHLH) TFs and WD-repeat proteins (Jaakola, 2013).

In strawberry, overexpression of *FaMYB10* resulted elevated levels of anthocyanin in the leaves, flowers, fruits, and roots (Lin-Wang et al., 2010, 2014). Recent studies suggest hormonal regulation of *MYB* expression: during ripening auxin negatively, but ABA positively regulates the expression of *FaMYB10* in strawberry receptacles (Medina-Puche et al., 2014). In contrast to strawberry, overexpression of *FaMYB1* in tobacco suppressed the accumulation of anthocyanin and flavonol by repression of tobacco homolog (Aharoni et al., 2001). MYB regulates the flavonoid levels in capsicum, Chinese bayberry and grape by up-regulating flavonoid biosynthetic genes encoding chalcone synthase (CHS), chalcone isomerase, flavanone 3-hydroxylase (F3H), flavonoid 30-hydroxylase (F30H), dihydroflavonol 4-reductase (DFR), anthocyanidin synthase (ANS) and UDP glucose: flavonoid 3-*O*-glucosyltransferase (UFGT) (Walker et al., 2007; Niu et al., 2010; Li et al., 2011). In grapes, *VvMYBA1* and *VvMYBA2* regulates the anthocyanin content of berries, for instance, inactivation of *VvMYBA2* due to mutation in conserved domain results in white berries (Walker et al., 2007).

Normally the fruits of cultivated tomatoes do not accumulate high levels of anthocyanin in the peel or flesh. However, the peel of tomatoes wild relative *S. chilense* relatively accumulate high levels of anthocyanin under control of MYB family transcription factor *anyhocynin1* (ANT1) (Mathews et al., 2003). Schreiber et al. (2012) showed overexpression of *S. chilense ANT1* and *ANT2* in tomato cultivar exceptionally increased the levels of flavonoids in the cotyledon, leaves, floral organ and fruit peel. Additionally, the accumulation of flavonoid such as naringenin chalcone in the tomato fruit peel is controlled by *SLMYB12*, downregulation of it results colourless peel phenotype in *yellow* mutant, which was rescued by overexpression of *MYB12* (Schreiber et al., 2012). In tomato, the green shoulder at the top end is very common in wild type, but this phenotype lacks in *uniform ripening* (*u*) mutant. The *U* gene encodes for protein GOLDEN2-LIKE (GLK); a transcription factor, GARP subfamily of the MYB super family. Interestingly, overexpression of either *SLGLK1* or *SLGLK2* in both *u* and U background Ailsa Craig mimicked *hp* fruit phenotype such as enhanced chloroplast and high carotenoids (Nguyen et al., 2014).

### Improving Fruit Shelf Life

The initial evidence of ethylene in ripening led researchers to target genes such as *ACS* and *ACO*, which were involved in ethylene biosynthesis. The antisense *ACS* and *ACO* transgenic plants produce non-climacteric tomato fruits which ripen in presence of external ethylene (Hamilton et al., 1990; Oeller et al., 1991; Picton et al., 1993). Similar results were obtained in other agronomic crops transgenic like melon and papaya (Kumar et al., 2014). Later metabolite SAM and ACC, precursors of ethylene biosynthesis was targeted to achieve delayed ripening. SAM methyltransferases regulates the levels of SAM, which methylate homocysteine to methionine. SAM hydroxylase breaks SAM to methyl thioadenosine and homoserine instead of *S*-adenosyl-L-homocysteine thus resulting in low level of SAM. Good et al. (1994) demonstrated the expression of the SAM hydroxylase in tomatoes fruit results reduced levels of ethylene and delayed ripening. On the other hand, the expression of prokaryotic ACC deaminase in tomato plant effectively decreases the available cellular ACC, which facilitates ethylene formation. The bacterial ACC deaminase protein is able to reverse the breakdown of ACC into α-ketobutyric acid and ammonia. Transgene expression of ACC deaminase enzyme in tomato resulted reduced ethylene levels that delayed ripening and enhanced post-harvest life (Klee et al., 1991). In apple, disruption of *MdACS3* gene using a transposon-tagging technique confers prolonged shelf life of fruits (Wang et al., 2009).

Respiration has huge impact on fruit shelf life. To elucidate the role of respiration on fruit texture and post-harvest sustainability, the levels of Krebs cycle intermediates were manipulated in tomato. The recent report of Centeno et al. (2011) revealed that malate and fumarate plays significant role in the post-harvest transpirational water loss. The upregulation of both malate and fumarate in the transgenic of tomato antisense *Malate dehydrogenase* (*MDH*) resulted enhanced post-harvest shelf life due to decreased amount of post-harvest transpirational water loss (Centeno et al., 2011).

### Metabolic Engineering of Phytohormones to Improve Quality and Stress Tolerance

#### Improving Quality

The growth and development of plant is facilitated by hormones. The function of phytohormones in the tissue and organ differentiation was evident from the hybrids in Arabidopsis (C24/Col), where increased IAA level enhanced leaf cell numbers and reduced salicylic acid (SA) level promoted size of photosynthetic cells (Groszmann et al., 2015). The overexpression of Brassica gene shoot meristemless (STM) in Arabidopsis reduced the level of abscisic acid (ABA) and cytokinins, caused an enhanced growth of SAM and the ectopic meristem, which eventually reflected as lobed leaves, and increased number of reproductive organs such as flowers and siliques (Elhiti and Stasolla, 2012).

A recent work suggests cross talk between hormones during growth and development (Kumar et al., 2014). In tomato, inhibition of *AUXIN RESPONSE FACTOR* 7 (*SlARF*7) can produce seedless parthenocarpic fruits (De Jong et al., 2009; De Jong et al., 2011). Similarly, suppression of *ARF4* and *GH3* genes, combined with high ethylene production in *AP2a* suppressed transgenic lines suggest ethylene mediated response of auxin (Karlova et al., 2011). Interestingly, non-climacteric fruits, such as grape and citrus are much more dependent on the ABA (Setha, 2012).

In tomato, suppression of ABA biosynthetic gene *9-cis-epoxycarotenoid dioxygenase1* (*NCED1*) results non-climacteric pattern of ripening due to low levels of ethylene (Sun et al., 2012a). In fact, ABA negatively regulates carotenoid levels in fruits. For example, ABA deficiency in *hp3, flc* and *sit* mutants of tomato causes over-pigmentation in fruits (Galpaz et al., 2008). Similar phenotype was evident in *SlNCED1* silenced transgenic tomato fruits, which accumulates high levels of lycopene and β-carotene (Sun et al., 2012a). In banana, ABA in coordination with ethylene promotes cell wall hydrolysis and fruit softening (Lohani et al., 2004), whereas in grapes ABA promotes fruit colouration and softening (Cantín et al., 2007). In non-climacteric fruits such as strawberry and grapes, ABA influences the flavonoid content, but in the ethylene dependent manner, because application of 1-methylcyclopropene (MCP, an ethylene inhibitor) delays anthocyanin accumulation. The role of ABA in flavonoid biosynthesis was confirmed through the rescue of colourless phenotype in *NCED* silenced strawberry fruits after exogenous ABA treatment (Jia et al., 2011). Likewise, methyl jasmonate enhances anthocyanin accumulation in the strawberry fruit peel by up-regulating phenyl-propanoid pathway related genes (*CHS, DFR, UFGT, PAL1, C4H, CHI* and *F3H*). In addition, MeJA and SA are also known to be involved in fruit softening (Srivastava and Dwivedi, 2000; Concha et al., 2013). Recent work of Liu et al. (2012) provides direct evidences for the role of jasmonate in carotenoid biosynthesis. The jasmonate deficiency in the tomato mutants *def1* (defective in the octadecanoid synthesis pathway) and *spr2* (s*uppressor of pro-systemin-mediated responses2*) reduces the lycopene content due to downregulation of carotenogenesis (Liu et al., 2012).

#### Response to Stress

Mass spectrometry based plant metabolomics has geared up the evaluation of metabolite responses to stress (Jorge et al., 2016; Tenenboim and Brotman, 2016). For instance, ABA is recognized as the stress response hormone that signals shoot for anti-transpirant activities such as reduction of leaf size and stomatal closure during water deficit condition (Wilkinson and Davies, 2002; Davies et al., 2005) and facilitates deeper root growth by altering root architecture under scarcity of water and nitrogen deficiency (Spollen et al., 2000). ABA mediated drought tolerance in plants involves modulation of root aquaporins, and enhanced cell turgor pressure management by affecting the biosynthesis of antioxidant enzymes and soluble solutes (Chaves et al., 2003; Parent et al., 2009). The over expressing *NCED1* gene in tomato leading to stomatal closure during water deficiency confers tolerance against drought (Thompson et al., 2007). However, the increased stomatal closure in the *NCED1* over expressing transgenic line affects the overall carbon assimilation, which exerts a dramatic influence on the number of seeds. Therefore, the repercussions of the ABA-induced drought resistant in plant includes reduced crop yield, sterile pollen and seed dormancy (due to elevated levels of ABA) (Ji et al., 2011). As a remedy, use of drought inducible gene (*ABA3*/*LOS5*, in rice) and promoter (*era1*, in canola) increases the ABA level along with the crop yield.

The growth hormone cytokinin (CK), acting antagonistically to the senescence hormone ABA, and promotes proliferation and differentiation of cell or tissue, thus preventing premature senescence. Agronomic trait stay green (enhanced the photosynthetic activity) in drought tolerant genotypes allows accumulation of higher levels of CK in tissue and xylem sap. This CK accumulation promotes normal grain filling and limits premature leaf senescence (Borrell et al., 2000). Researches have used the CK biosynthetic gene *isopentenyl transferase* (*ipt*) for improving crop performance under drought stress. To date, *ipt* gene has been tested in many crop species such as rice, pea, tobacco and cassava for high yield under reduced irrigation (Qin et al., 2011). The grain productivity in barley and rice was reported to improve under limited water supply through enhancing CK content by attenuation of *cytokinin oxidase* gene (Ashikari et al., 2005; Zalewski et al., 2010).

Brassinosteroids (BRs) are new class of phytohormones that regulate a wide range of bio-physiological activities such as plant growth, root development, flowering and reproduction, seed germination, and biotic and abiotic responses. Arabidopsis was widely used to study the genotype to phenotype correlation in the BRs biosynthetic or signaling mutants. For example, the BRs mutants exhibit hypersensitivity to the seed germination inhibition exerted by the ABA, and the exogenous application of BRs rescues the low seed germination phenotype in gibberellin (GA) mutant (Vriet et al., 2012). Overexpression of *hydroxysteroid dehydrogenase1* (*HSD1*, encodes a putative enzyme in BRs synthesis) gene in Arabidopsis resulted reduced seed dormancy compared to wild type (Baud et al., 2009). Similarly, in Arabidopsis overexpression of *DWARF4* (*DWF4*) gene rescued the ABA seed inhibition phenotype (Divi and Krishna, 2009). Interestingly, the overexpression of *DRAWF4/CYP90B1* gene in crop plants such as rice registered a positive response with respect to agronomical traits. The overexpression *DRAWF4/CYP90B1* transgenic rice showed increased CO_2_ uptake and enhanced photosynthetic efficiency, which increased the seed yield (Sakamoto and Matsuoka, 2008; Wu et al., 2008). The *CYP85A2* (encodes BRs biosynthetic enzymes) mutant confirmed the role of BRs in reproduction. The *cyp85a2* mutant exhibited phenotype similar to Arabidopsis mutant *seuss* because it lacks proper development of reproductive organs like ovule (Nole-Wilson et al., 2010). Furthermore, the downregulation of BRs in the maize *nana plant1* and *dwarf brassinosteroid-dependent1* (*brd1*) mutants result minimized male flowers (Hartwig et al., 2011; Makarevitch et al., 2012). Recently, disruption of *squalene synthase* (*SQS*) gene in rice by RNA-interference reduced the overall sterol content, including BRs, which reduced the stomatal conductance to provide drought tolerance during vegetative and reproductive stages (Manavalan et al., 2012). Apart from abiotic stress, BRs provides resistance against a broad range of diseases in potato, rape seed, rice, tomato and tobacco (Vriet et al., 2012). For example, the elevated levels of BRs in *Brassica juncea* improves the resistance against potent fungal pathogen *Botrytis cinerea* (Wang et al., 2012).

5-hydroxy tryptamine (serotonin) acts as neurotransmitter in animal system (Seo et al., 2008). Moreover, in plants, serotonin assumed as intermediate between tryptamine and IAA during auxin biosynthesis, but still more work and evidence is required to approve this hypothesis (Tivendale et al., 2010). Recently, the role of serotonin in senescence was demonstrated in rice leaf tissue (Kang et al., 2009). During senescence, leaf tissue synthesizes and accumulates high levels of serotonin to maintain cellular integrity. Additionally, inhibition of serotonin biosynthesis causes early senescence of leaf (Kang et al., 2009). Hence, serotonin could be used as a potential marker for senescence.

### Biofortification Enabled Nutrient Enrichment of Crops

#### Enhancing the Level of Provitamin A

The deficiency of vitamin A causes night blindness, which can further result in complete blindness. Interestingly, β-carotene acts as pro-vitamin A, and it was targeted to reduce the deficiency of vitamin A. Considering rice as one of the major staple food especially in the Asian region; the supplementation of vitamin A via β-carotene was initiated by enrichment of rice endosperm to produce golden rice (Ye et al., 2000; Paine et al., 2005). This approach involved, upregulation of carotenoid biosynthetic pathways in rice endosperm, which includes transgene expression of *phytoene synthase* (*psy*, from daffodil and maize) and *phytoene desaturase* (*crt1*, from *Erwinia uridovora*) under endosperm specific Glutelin (Gt1). This resulted in an increase of up to 27 fold (37 μg/g) in the β-carotene levels in golden rice. Interestingly, transgene overexpression of three bacterial carotenoids biosynthetic genes *CrtB*, *CrtI*, and *CrtY*, encoding phytoene synthase, phytoene desaturase, and lycopene β-cyclase, respectively, resulted ∼40 fold increase of the β-carotene and ∼100–200 fold increase for total carotenoid (Diretto et al., 2010). Till date, several attempts have been made toward enrichment of β-carotene in important staple crop food species such as cassava, maize, potato and sweet potato (Martin et al., 2011; Tan and Zhao, 2017).

#### Enhancing the Level of Folates

Folates belongs to the class of vitamin B, act as Co-factors for C_1_-metabolism (one-carbon transfer reactions) such as amino acid metabolism, nucleotide biosynthesis and the methylation cycle (Hanson and Roje, 2001). Deficiency of folate in human causes birth defect, increases cardiovascular disease and megaloblastic anemia. Plants are capable of biosynthesizing folate in mitochondria and plastids from pterins. Pterins are synthesized from guanosine-50- triphosphate (GTP) and *p*-aminobenzoate (PABA) (Hanson and Roje, 2001). The overexpression of folate biosynthetic gene *GTP-cyclohydrolase 1* (*GTPCH1*) in transgenic tomato enhanced the pterins content of ripe fruits, which resulted two-fold increase in the folate content (Díaz de la Garza et al., 2004). Recently, a transgenic lettuce expressing synthetic codon-optimized gene *GTPCH1* was generated, which had showed 2.1–8.5 fold higher levels of folate compared to non-transgenic plant (Nunes et al., 2009). However, previous attempts suggest 100 times increased folate content in the overexpression transgenic rice, which contains two transgenes from Arabidopsis, *GTPCH1* and *aminodeoxychorismate synthase* (*ADCS*) (Storozhenko et al., 2007; Dong et al., 2014). Naqvi et al. (2009) generated an elite inbred of transgenic Maize, in which, the kernel endosperm contained double amount of folate, six fold of ascorbate and 169 fold of β-carotene. Interestingly, they used four genes from different sources; *PSY1* from maize under glutenin promoter and *CRT1* from *Pantoea ananatis*, *GTPCH1* from *Escherichia coli* and *dehydroascorbate reductase* (*DHAR*) from rice under barley D-hordein promoter (Naqvi et al., 2009). Notably, the above mentioned leads are crop/genotype dependent, because transgenic lines of potato and Arabidopsis failed to accumulate higher levels of folate (Blancquaert et al., 2013). Hence, a better understanding of folate pathway is required, which could be useful and applicable to enhance the folates content in wide range of plant species.

#### Altering the Levels of Flavonoids

Flavonoids play an important role in the maintenance of fruit quality. It represents a huge family of secondary metabolites that consists of more than 6000 compounds (Hichri et al., 2011). The peel of fleshy fruits like grape and strawberry accumulates flavonoids such as anthocyanin, catechin, epicatechin, quercetin, kaempferol, myricetin, and isorhamnetin. Butelli et al. (2008), ectopically expressed the *Del*/*Ros1*gene (from snapdragon plant) in tomato under fruit specific E8 promoter. As a result, the fruits of *Del*/*Ros1* tomato transgenic lines accumulated substantial amount of anthocyanin (lycopene is the major secondary metabolite in cultivated tomatoes) due to the increased expression of anthocyanin biosynthetic genes (Butelli et al., 2008). Recently, the genetically engineered purple tomato was investigated to demonstrate the impact of anthocyanin (antioxidant) on prolonging fruit shelf life and resistance against fungal infection (Zhang et al., 2013b).

#### Altering Flavor and Aroma

The flavor and aroma of fruit are important and it influences the customer choices. Over the past decades, most of the research on fruit and vegetable crop species was mainly focused on the yield and resistance. Genome wide association mapping and metabolite assisted quantitative trait loci analysis has helped to fish out useful genes that confers aroma of rice grain (Daygon, 2016). The recent advancement in the field of metabolomics and the available metabolic network databases has fascinated researcher to focus on flavor and aroma. Breeding has long served toward improvement of flavor; however, it was dedicated more toward a balance between sugar: organic acid ratio and the post-harvest management (Jones and Scott, 1983). Recent studies on flavor and aroma include metabolic engineered tomato. The heterologous expression of *Clarkia breweri* plant S*-linalool synthase* (*LIS*) gene in tomato resulted accumulation of *S-*linalool and 8-hydroxylinalool at the ripe stage of fruit (Lewinsohn et al., 2001). In addition to *S*-linalool, the transgenic exhibited increased levels of geranial, limonene, myrcene, and β-ocimene, and a decrease in nor-isoprenes. Similarly, the metabolic analysis of fruits from the overexpression lines of tomato *alpha-Zingiberene synthase* (*ZIS*, encodes for sesquiterpene synthase) transgenic showed higher levels of alpha-zingiberene and other sesquiterpenes, such as 7-epi-sesquithujene, alpha-bergamotene, beta-bisabolene and beta-curcumene, whereas control fruit showed absence of sesquiterpenes (Davidovich-Rikanati et al., 2008). Zawirska-Wojtasiak et al. (2009) studied the aroma in transgenic cucumber. The GC/MS based study of transgenic cucumber expressing transgene preprothaumatin II gene under 35S promoter showed enhanced production of (E, Z)-2,6-nonadienal (Zawirska-Wojtasiak et al., 2009).

Fragrance of flowers is known to play multiple roles including attraction of pollinators and the interaction between plant and their surroundings. The ornamental plants like lisianthus (*Eustoma grandiflorum*) produces beautiful flowers, but these lacks floral scent (Aranovich et al., 2007). Transformation of lisianthus with *benzyl alcohol acetyltransferase* (*BEAT;* obtained from *Clarkia breweri*) under constitutive CaMV 35S promoter generated substrate dependent transgenic which produced 5–7 times higher levels of benzyl acetate (aromatic compound) when treated with benzyl alcohol (Aranovich et al., 2007). Interestingly, the recent report displayed the role of the aroma profile in stress tolerance. For example, *omega-3 fatty acid desaturases FAD3* and *FAD7* genes (involves in the conversion of C18:2 to C18:3, a precursor for hexanals and its derivatives) were over expressed in tomato to achieve cold stress tolerance (Domínguez et al., 2010). The overexpression transgenic tomato exhibited increased levels of 18:3/18:2 and (Z)-hex-3-enal/hexanal ratio with enhanced cold stress tolerance. Similarly, ectopic expression of aroma biosynthetic transgenes, such as strawberry *linalool/nerolidol synthase* (*FaNES1*) in Arabidopsis and potato, maize *terpene synthase* (*TPS10*) gene in potato, and *patchoulol synthase* (*PTS*) coupled with FPP synthase in tobacco was used to improve defense management from plant pest (Dudareva and Pichersky, 2008).

### Metabolomics to Cater Biofuel Demand

Burgeoning petroleum demand worldwide motivates researchers to explore renewable and alternative sources, such as biodiesel. In the current omics era, a refined understanding of biochemical pathways is being used to genetically improve biodiesel crop species, including jatropha, soybean, mustard, pongamia, algae, etc. The oil composition of the plant determines its quality. Currently, agronomical suitable jatropha (*Jatropha curcas*) is extensively grown as an alternative source of energy (Kumar et al., 2015). The oil content of jatropha seed is rich in polyunsaturated fatty acid mainly linoleic acid, which is vulnerable to oxidation and has negative impact on the quality. Silencing of *fatty acid desaturase* (*FAD2s*) in jatropha by Qu and colleagues significantly lowered the level of linoleic acid, while increasing the oleic acid content by 78%. To improve the yield and oil quality of jatropha under water deficient condition, three transgenics were raised by overexpressing genes *GSMT* and *DMT* (encodes enzyme catalyzes glycine betaine catalyses), *PPAT* (encodes an enzyme that catalyzes CoA biosynthetic pathway) and *NF-YBI* (encodes transcription factor NF-Y subunit) (Tsuchimoto et al., 2012). A range of candidate genes have been identified and characterized so far in jatropha, which are either involved in the metabolism of fatty acid or contribute to improve the seed oil content during stress. These genes include *PIP2* encoding aquaporin protein, *betaine aldehyde dehydrogenase*, *Δ6-fatty acid desaturase*, *ω6-fatty acid desaturase*, *ω3-fatty acid desaturase*, *diacylglycerol acyltransferase* and *long chain acyl coenzyme A synthetase* (Kumar et al., 2015). Similar to jatropha, castor is another excellent source of biodiesel because of its transesterified oil, which is soluble in alcohol without heating (Sujatha et al., 2008). Castor yield was improved by overexpressing the *Cry1Ab* gene, which provides resistance against feeders (Malathi et al., 2006). A non-transgenic method targeting induced local lesions in genomes (TILLING) was also used to increase the quality of caster seed oil by knocking out the gene that encodes ricin (alkaloid inhibits protein synthesis)^4^. The use of seed oil as biodiesel has been extended to many plant species such as coconut, cotton, mustard, pongamia, sunflower, etc. (Vaughn et al., 2009; Dwivedi et al., 2011; Lafont et al., 2015; Ortiz-Martínez et al., 2016; Saydut et al., 2016).

Photosynthetic water micro algae are also a rich source of oils that are mainly composed of unsaturated fatty acids. The simple cellular structure of these microorganisms, and their only dependency on CO_2_, water and the sunlight for their rapid growth renders algal derived biodiesel more accessible than that obtained from higher plants. Attempts aiming at genetic manipulation of algae were undertaken to increase the oil content (Beer et al., 2009). The overexpression of *DGAT* (gene from the fatty acid biosynthetic pathway) in *Chlamydomonas reinhardtii*, however, produced unintended result, i.e., no increase for total fatty acid content (La Russa et al., 2012).

Biofuels are also obtained through fermentation of sugars yielding alcohol such as ethanol and butanol. In sugarcane, the load of sucrose, trehalulose and isomaltulose was obtained by overexpressing trehalulose synthase and sucrose isomerase (Wu and Birch, 2007; Hamerli and Birch, 2011; Arruda, 2012). Similarly, xylanases from bacterial and fungal source was expressed in crop plants such as barley, potato, rice, sunflower, and tobacco (Willis et al., 2016). Xylanases degrade β-1,4-xylan to pentose sugar, which can easily be fermented to alcohols. In addition, several other hydrolases (endo-Glucanases, cellobiohydrolases, β-glucosidases, glycosyl hydrolase etc.) were overexpressed to improve the biofuel availability from plants (Willis et al., 2016).

## Metabolomics: An Integral Part of Knowledge-Based Plant Breeding

The last decade has witnessed tremendous advancements in technologies followed by their deployment in understanding different facets of biology to understand the complex biology of desired traits. Knowledge-based plant breeding (KPB) utilizes all the meaningful information inferred from analysis of the large-scale data pertaining to genome, epigenome, transcriptome, metabolome, and proteome that collectively lead to a particular phenotype. The data generated from these ‘Omics’ approaches enable better understanding of the systems biology of the trait, so far mostly contributed by systems genetics and genomics, and to some extent transcriptomics. The other ‘Omics’ approaches, including metabolomics and proteomics have also started contributing toward generating important information, which can become an integral component of KPB, thereby strengthening the approach further for achieving higher genetic gain.

Technological advancement has contributed to improve the efficiency of plant breeding techniques via precise selection of desired plants. An easy access to the different ‘Omics’ platforms will cause a paradigm shift in breeding process by facilitating plant selections based on the genome-scale information generated at different levels of biological processes. The plant breeders will gradually embrace these developments, which in turn will help them to make informed decisions.

## Concluding Remarks and Future Perspectives

Advances in plant metabolomics in recent time has allowed the precise selection of desirable traits along with offering opportunities to undertake metabolic engineered plants. The shift of technology from single metabolite analysis to high throughput assays generating footprints of a variety of metabolites in one go has paved the way for discovery/construction of better models for metabolite networks, and the identification of robust biomarkers. In the last decade, the implementation of metabolomics in conjunction with other omics technologies has not only uncovered a plethora of known as well as novel metabolites, but also allowed to determine their specific contribution toward improving key plant attributes such as quality, yield, shelf life, etc. To this end, high throughput genotyping/sequencing platforms based on NGS technology has been a tremendous support as a cost-effective and high-throughput means to elucidate the architecture of metabolic traits. The newly created avenues such as GWAS, GS and EWAS that allow efficient integration of metabolite profiling could provide a great impetus to metabolomics assisted breeding. We anticipate that the integration of metabolomics and the other omics tools greatly improves the ability of a plant breeder in order to design and develop agronomically superior plants, thus enabling rapid development of high-performing crop genotypes that adequately meet the challenges of 21st century agriculture.

## Author Contributions

RK, AB, MKP, and AK designed the article. RK and AK wrote the article. AB, AP, RK, and MKP corrected the article and finally all authors read and approved the final article.

## Conflict of Interest Statement

The authors declare that the research was conducted in the absence of any commercial or financial relationships that could be construed as a potential conflict of interest.

## Footnotes

1 https://www.arabidopsis.org/biocyc/

2 http://www.plantcyc.org/

3 https://www.ipcc.ch/pdf/assessment-report/ar5/wg1/WGIAR5_SPM_brochure_en.pdf

4 www.arcadiabio.com

## References

[B1] Adjei-Afriyie F. Kim C. S. Takemura M. Ishikawa M. Horiike M. (2000). Isolation and identification of the probing stimulants in the rice plant for the white-back planthopper, *Sogatella furcifera* (Homoptera: Delphacidae). *Biosci. Biotechnol. Biochem.* 64 443–446. 10.1271/bbb.64.443 10737209

[B2] Afendi F. M. Okada T. Yamazaki M. Hirai-Morita A. Nakamura Y. Nakamura K. (2012). KNApSAcK family databases: integrated metabolite-plant species databases for multifaceted plant research. *Plant Cell Physiol.* 53 e1 10.1093/pcp/pcr165 22123792

[B3] Aharoni A. De Vos C. H. Wein M. Sun Z. Greco R. Kroon A. (2001). The strawberry FaMYB1 transcription factor suppresses anthocyanin and flavonol accumulation in transgenic tobacco. *Plant J.* 28 319–332. 10.1046/j.1365-313X.2001.01154.x 11722774

[B4] Aharoni A. Giri A. P. Verstappen F. W. A. Bertea C. M. Sevenier R. Sun Z. (2004). Gain and loss of fruit flavor compounds produced by wild and cultivated strawberry species. *Plant Cell* 16 3110–3131. 10.1105/tpc.104.023895 15522848 PMC527202

[B5] Ainalidou A. Tanou G. Belghazi M. Samiotaki M. Diamantidis G. Molassiotis A. (2015). Integrated analysis of metabolites and proteins reveal aspects of the tissue-specific function of synthetic cytokinin in kiwifruit development and ripening. *J. Proteomics* 143 318–333. 10.1016/j.jprot.2016.02.013 26915585

[B6] Albacete A. A. Martínez-Andújar C. Pérez-Alfocea F. (2014). Hormonal and metabolic regulation of source-sink relations under salinity and drought: from plant survival to crop yield stability. *Biotechnol. Adv.* 32 12–30. 10.1016/j.biotechadv.2013.10.005 24513173

[B7] Alhagdow M. Mounet F. Gilbert L. Nunes-Nesi A. Garcia V. Just D. (2007). Silencing of the mitochondrial ascorbate synthesizing enzyme L-galactono-14-lactone dehydrogenase affects plant and fruit development in tomato. *Plant Physiol.* 145 1408–1422. 10.1104/pp.107.106500 17921340 PMC2151702

[B8] Aranovich D. Lewinsohn E. Zaccai M. (2007). Post-harvest enhancement of aroma in transgenic lisianthus (*Eustoma grandiflorum*) using the *Clarkia breweri* benzyl alcohol acetyltransferase (BEAT) gene. *Postharvest Biol. Technol.* 43 255–260. 10.1016/j.postharvbio.2006.09.001

[B9] Arora V. K. Boer G. J. (2014). Terrestrial ecosystems response to future changes in climate and atmospheric CO_2_ concentration. *Biogeosciences* 11 4157–4171. 10.5194/bg-11-4157-2014

[B10] Arruda P. (2012). Genetically modified sugarcane for bioenergy generation. *Curr. Opin. Biotechnol.* 23 315–322. 10.1016/j.copbio.2011.10.012 22093808

[B11] Ashikari M. Sakakibara H. Lin S. Yamamoto T. Takashi T. Nishimura A. (2005). Cytokinin oxidase regulates rice grain production. *Science* 309 741–745. 10.1126/science.1113373 15976269

[B12] Azzi L. Deluche C. Gévaudant F. Frangne N. Delmas F. Hernould M. (2015). Fruit growth-related genes in tomato. *J. Exp. Bot.* 66 1075–1086. 10.1093/jxb/eru527 25573859

[B13] Baldet P. Hernould M. Laporte F. Mounet F. Just D. Mouras A. (2006). The expression of cell proliferation-related genes in early developing flowers is affected by a fruit load reduction in tomato plants. *J. Exp. Bot.* 57 961–970. 10.1093/jxb/erj082 16488916

[B14] Ban Y. Honda C. Hatsuyama Y. Igarashi M. Bessho H. Moriguchi T. (2007). Isolation and functional analysis of a MYB transcription factor gene that is a key regulator for the development of red coloration in apple skin. *Plant Cell Physiol.* 48 958–970. 10.1093/pcp/pcm066 17526919

[B15] Barros E. Lezar S. Anttonen M. J. van Dijk J. P. Röhlig R. M. Kok E. J. (2010). Comparison of two GM maize varieties with a near-isogenic non-GM variety using transcriptomics, proteomics and metabolomics. *Plant Biotechnol. J.* 8 436–451. 10.1111/j.1467-7652.2009.00487.x 20132517

[B16] Baud S. Dichow N. R. Kelemen Z. d’Andréa S. To A. Berger N. (2009). Regulation of HSD1 in seeds of *Arabidopsis thaliana*. *Plant Cell Physiol.* 50 1463–1478. 10.1093/pcp/pcp092 19542545

[B17] Beer L. L. Boyd E. S. Peters J. W. Posewitz M. C. (2009). Engineering algae for biohydrogen and biofuel production. *Curr. Opin. Biotechnol.* 20 264–271. 10.1016/j.copbio.2009.06.002 19560336

[B18] Bénard C. Bernillon S. Biais B. Osorio S. Maucourt M. Ballias P., (2015). Metabolomic profiling in tomato reveals diel compositional changes in fruit affected by source–sink relationships. *J. Exp. Bot.* 66 3391–3404. 10.1093/jxb/erv151 25873655 PMC4449552

[B19] Bentsink L. Alonso-Blanco C. Vreugdenhil D. Tesnier K. Groot S. P. Koornneef M. (2000). Genetic analysis of seed-soluble oligosaccharides in relation to seed storability of Arabidopsis. *Plant Physiol.* 124 1595–1604. 10.1104/pp.124.4.1595 11115877 PMC59858

[B20] Bernillon S. Biais B. Deborde C. Maucourt M. Cabasson C. Gibon Y. (2013). Metabolomic and elemental profiling of melon fruit quality as affected by genotype and environment. *Metabolomics* 9 57–77. 10.1007/s11306-012-0429-1

[B21] Bertin N. Gautier H. Roche C. (2002). Number of cells in tomato fruit depending on fruit position and source-sink balance during plant development. *Plant Growth Regul.* 36 105–112. 10.1023/A:1015075821976

[B22] Bessire M. Chassot C. Jacquat A. C. Humphry M. Borel S. Petétot J. M. (2007). A permeable cuticle in *Arabidopsis* leads to a strong resistance to Botrytis cinerea. *EMBO J.* 26 2158–2168. 10.1038/sj.emboj.7601658 17396154 PMC1852784

[B23] Bird A. (2007). Perceptions of epigenetics. *Nature* 447 396–398. 10.1038/nature05913 17522671

[B24] Blancquaert D. Storozhenko S. Van Daele J. Stove C. Visser R. G. Lambert W. (2013). Enhancing pterin and para-aminobenzoate content is not sufficient to successfully biofortify potato tubers and *Arabidopsis thaliana* plants with folate. *J. Exp. Bot.* 64 3899–3909. 10.1093/jxb/ert224 23956417

[B25] Bloom A. Burger M. Kimball B. Pinter P. J. (2014). Nitrate assimilation is inhibited by elevated CO_2_ in field-grown wheat. *Nat. Clim. Change* 4 477–480. 10.1038/nclimate2183

[B26] Bloom A. J. Asensio J. S. R. Randall L. Rachmilevitch S. Cousins A. B. Carlisle E. A. (2012). CO_2_ enrichment inhibits shoot nitrate assimilation in C_3_ but not C_4_ plants and slows growth under nitrate in C_3_ plants. *Ecology* 93 355–367. 10.1890/11-0485.1 22624317

[B27] Bohner J. Ban F. (1988). Cell number, cell size and hormone levels in semi-isogenic mutants of *Lycopersicon pimpinellifolium* differing in fruit size. *Cell* 72 316–320. 10.1111/j.1399-3054.1988.tb05839.x

[B28] Bohra A. Singh N. P. (2015). Whole genome sequences in pulse crops: a global community resource to expedite translational genomics and knowledge-based crop improvement. *Biotechnol. Lett.* 37 1529–1539. 10.1007/s10529-015-1836-y 25851953

[B29] Borrell A. K. Hammer G. L. Douglas A. C. L. (2000). Does maintaining green leaf area in sorghum improve yield under drought? I. Leaf growth and senescence. *Crop Sci.* 40 1026–1037. 10.2135/cropsci2000.4041026x

[B30] Boyer J. Liu R. H. (2004). Apple phytochemicals and their health benefits. *Nutr. J.* 3:5 10.1186/1475-2891-3-5 PMC442131 15140261

[B31] Brown S. C. Kruppa G. Dasseux J. L. (2005). Metabolomics applications of FT-ICR mass spectrometry. *Mass Spectrom. Rev.* 24 223–231. 10.1002/mas.20011 15389859

[B32] Burgos A. Szymanski J. Seiwert B. Degenkolbe T. Hannah M. A. Giavalisco P. (2011). Analysis of short-term changes in the *Arabidopsis thaliana* glycerolipidome in response to temperature and light. *Plant J.* 66 656–668. 10.1111/j.1365-313X.2011.04531.x 21309866

[B33] Butelli E. Titta L. Giorgio M. Mock H. P. Matros A. Peterek S. (2008). Enrichment of tomato fruit with health-promoting anthocyanins by expression of select transcription factors. *Nat. Biotechnol.* 26 1301–1308. 10.1038/nbt.1506 18953354

[B34] Buxdorf K. Rubinsky G. Barda O. Burdman S. Aharoni A. Levy M. (2014). The transcription factor SlSHINE3 modulates defense responses in tomato plants. *Plant Mol. Biol.* 84 37–47. 10.1007/s11103-013-0117-1 23943056

[B35] Caasi-Lit M. T. Tanner G. J. Nayudu M. Whitecross M. I. (2007). Isovitexin–2’-O-beta [6-O-E-*p*-coumaroylglucopyranoside] from UV-B irradiated leaves of rice, *Oryza sativa* L. inhibits fertility of *Helicoverpa armigera*. *Photochem. Photobiol.* 83 1167–1173. 10.1111/j.1751-1097.2007.00125.x 17880511

[B36] Cai C. Yin X. He S. Jiang W. Si C. Struik P. C. (2016). Responses of wheat and rice to factorial combinations of ambient and elevated CO_2_ and temperature in FACE experiments. *Glob. Change Biol.* 22 856–874. 10.1111/gcb.13065 26279285

[B37] Cantín C. M. Fidelibus M. W. Crisosto C. H. (2007). Application of abscisic acid (ABA) at veraison advanced red color development and maintained post-harvest quality of “Crimson Seedless” grapes. *Postharvest Biol. Technol.* 46 237–241. 10.1016/j.postharvbio.2007.05.017

[B38] Carrari F. Baxter C. Usadel B. Urbanczyk-Wochniak E. Zanor M. I. Nunes-Nesi A. (2006). Integrated analysis of metabolite and transcript levels reveals the metabolic shifts that underlie tomato fruit development and highlight regulatory aspects of metabolic network behavior. *Plant Physiol.* 142 1380–1396. 10.1104/pp.106.088534 17071647 PMC1676044

[B39] Cebulj A. Cunja V. Mikulic-Petkovsek M. Veberic R. (2017). Importance of metabolite distribution in apple fruit. *Sci. Hortic.* 214 214–220. 10.1016/j.scienta.2016.11.048

[B40] Centeno D. C. Osorio S. Nunes-Nesi A. Bertolo A. L. Carneiro R. T. Araújo W. L. (2011). Malate plays a crucial role in starch metabolism, ripening, and soluble solid content of tomato fruit and affects post-harvest softening. *Plant Cell* 23 162–184. 10.1105/tpc.109.072231 21239646 PMC3051241

[B41] Chakraborty K. Uprety D. C. (2012). Elevated CO2 alters seed composition and quality in Brassica. *Indian J. Plant Physiol.* 17 84–87.

[B42] Chandler V. Alleman M. (2008). Paramutation: epigenetic instructions passed across generations. *Genetics* 178 1839–1844. 18430919 10.1093/genetics/178.4.1839 PMC2323780

[B43] Chassot C. Métraux J. P. (2005). The cuticle as source of signals for plant defense. *Plant Biosyst.* 139 28–31. 10.1146/annurev-phyto-080508-081820

[B44] Chassot C. Nawrath C. Métraux J. P. (2008). The cuticle: not only a barrier for plant defence: a novel defence syndrome in plants with cuticular defects. *Plant Signal. Behav.* 3 142–144. 10.4161/psb.3.2.5071 19704737 PMC2634007

[B45] Chaves M. M. Maroco J. P. Pereira J. S. (2003). Understanding plant responses to drought from genes to the whole plant. *Funct. Plant Biol.* 30 239–264. 10.1071/FP02076 32689007

[B46] Chawla G. Ranjan C. (2016). Principle, instrumentation, and applications of UPLC: a novel technique of liquid chromatography. *Open Chem. J.* 3 1–16. 10.2174/1874842201603010001

[B47] Chen W. Gao Y. Xie W. Gong L. Lu K. Wang W. (2014). Genome-wide association analyses provide genetic and biochemical insights into natural variation in rice metabolism. *Nat. Genet.* 46 714–721. 10.1038/ng.3007 24908251

[B48] Chen W. Wang W. Peng M. Gong L. Gao Y. Wan J. (2016). Comparative and parallel genome-wide association studies for metabolic and agronomic traits in cereals. *Nat. Commun.* 7:12767 10.1038/ncomms12767 PMC5059443 27698483

[B49] Cheng P. Walden D. B. (2005). Cuticle of maize (*Zea mays* L.) anther. *Microsc. Microanal.* 11 1152CD-1153CD 10.1104/pp.16.00629

[B50] Cho K. Cho K. Sohn H. Ha I. J. Hong S. (2016). Network analysis of the metabolome and transcriptome reveals novel regulation of potato pigmentation. *J. Exp. Bot.* 67 1519–1533. 10.1093/jxb/erv549 26733692 PMC4762390

[B51] Cho K. Shibato J. Agrawal G. K. Jung Y. Kubo A. (2008). Integrated transcriptomics, proteomics, and metabolomics analyses to survey ozone responses in the leaves of rice seedling. *J. Proteome Res.* 7 2980–2998. 10.1021/pr800128q 18517257

[B52] Concha C. M. Figueroa N. E. Poblete L. Oñate F. Schwab W. Figueroa C. R. (2013). Methyl jasmonate treatment induces changes in fruit ripening by modifying the expression of several ripening genes in *Fragaria chiloensis* fruit. *Plant Physiol. Biochem.* 70 433–444. 10.1016/j.plaphy.2013.06.008 23835361

[B53] Cuadros-Inostroza A. Ruíz-Lara S. González E. Eckardt A. Willmitzer L. Peña-Cortés H. (2016). GC–MS metabolic profiling of Cabernet Sauvignon and Merlot cultivars during grapevine berry development and network analysis reveals a stage- and cultivar-dependent connectivity of primary metabolites. *Metabolomics* 12 39 10.1007/s11306-015-0927-z PMC4723623 26848290

[B54] Cubas P. Vincent C. Coen E. (1999). An epigenetic mutation responsible for natural variation in floral symmetry. *Nature* 401 157–161. 10.1038/43657 10490023

[B55] Curvers K. Seifi H. Mouille G. de Rycke R. Asselbergh B. Van Hecke A. (2010). Abscisic acid deficiency causes changes in cuticle permeability and pectin composition that influence tomato resistance to *Botrytis cinerea*. *Plant Physiol.* 154 847–860. 10.1104/pp.110.158972 20709830 PMC2949027

[B56] Cuthbertson D. Andrews P. K. Reganold J. P. Davies N. M. Lange B. M. (2012). Utility of metabolomics toward assessing the metabolic basis of quality traits in apple fruit with an emphasis on antioxidants. *J. Agric. Food Chem.* 60 8552–8560. 10.1021/jf3031088 22881116 PMC3551554

[B57] Cuzzuol G. R. F. De Carvalho M. A. M. Zaidan L. B. P. (2005). Growth, photosynthate partitioning and fructan accumulation in plants of *Vernonia herbacea* (Vell.) Rusby under two nitrogen levels. *Braz. J. Plant Physiol.* 17 401–410. 10.1590/S1677-04202005000400008

[B58] D’Aoust M. A. Yelle S. Nguyen-Quoc B. (1999). Antisense inhibition of tomato fruit sucrose synthase decreases fruit setting and the sucrose unloading capacity of young fruit. *Plant Cell* 11 2407–2418. 10.1105/tpc.11.12.2407 10590167 PMC144148

[B59] Davidovich-Rikanati R. Lewinsohn E. Bar E. Iijima Y. Pichersky E. Sitrit Y. (2008). Overexpression of the lemon basil α-zingiberene synthase gene increases both mono- and sesquiterpene contents in tomato fruit. *Plant J.* 56 228–238. 10.1111/j.1365-313X.2008.03599.x 18643974

[B60] Davies W. J. Kudoyarova G. Hartung W. (2005). Long-distance ABA signaling and its relation to other signaling pathways in the detection of soil drying and the mediation of the plant’s response to drought. *J. Plant Growth Regul.* 24 285–295. 10.1007/s00344-005-0103-1

[B61] Daygon V. (2016). *Genome-wide Association Mapping of Aroma in Rice.* Doctoral thesis, The University of Queensland, Brisbane, QLD.

[B62] Daygon V. Fitzgerald M. (2013). “Application of metabolomics for providing a new generation of selection tools for crop improvement,” in *Hot Topics in Metabolomics: Food and Nutrition: Future Science Book Series* (London: Future Science Ltd), 6–16.

[B63] De Jong M. Mariani C. Vriezen W. H. (2009). The role of auxin and gibberellin in tomato fruit set. *J. Exp. Bot.* 60 1523–1532. 10.1093/jxb/erp094 19321650

[B64] De Jong M. Wolters-Arts M. García-Martínez J. L. Mariani C. Vriezen W. H. (2011). The *Solanum lycopersicum* AUXIN RESPONSE FACTOR 7 (SlARF7) mediates cross-talk between auxin and gibberellin signalling during tomato fruit set and development. *J. Exp. Bot.* 62 617–626. 10.1093/jxb/erq293 20937732 PMC3003806

[B65] De Palma M. Grillo S. Massarelli I. Costa A. Balogh G. Vigh L. (2008). Regulation of desaturase gene expression, changes in membrane lipid composition and freezing tolerance in potato plants. *Mol. Breed.* 21 15–26. 10.1007/s11032-007-9105-y

[B66] de Souza L. P. Naake T. Tohge T. Fernie A. R. (2017). From chromatogram to analyte to metabolite. How to pick horses for courses from the massive web-resources for mass spectral plant metabolomics. *Gigascience* 10.1093/gigascience/gix037 [Epub ahead of print]. PMC5499862 28520864

[B67] Degenkolbe T. Do P. T. Kopka J. Zuther E. Hincha D. K. Köhl K. I. (2013). Identification of drought tolerance markers in a diverse population of rice cultivars by expression and metabolite profiling. *PLoS ONE* 8:e63637 10.1371/journal.pone.0063637 PMC3661581 23717458

[B68] Degenkolbe T. Giavalisco P. Zuther E. Seiwert B. Hincha D. K. Willmitzer L. (2012). Differential remodeling of the lipidome during cold acclimation in natural accessions of *Arabidopsis thaliana*. *Plant J.* 72 972–982. 10.1111/tpj.12007 23061922

[B69] Della Vedova C. B. (2004). Paramutation: the chromatin connection. *Plant Cell* 16 1358–1364. 10.1105/tpc.160630 15178748 PMC490031

[B70] Desalegn G. Turetschek R. Kaul H. Wienkoop S. (2016). Microbial symbionts affect Pisum sativum proteome and metabolome under *Didymella pinodes* infection. *J. Proteomics* 143 173–187. 10.1016/j.jprot.2016.03.018 27016040

[B71] Díaz de la Garza R. Quinlivan E. P. Klaus S. M. J. Basset G. J. C. Gregory J. F. Hanson A. D. (2004). Folate biofortification in tomatoes by engineering the pteridine branch of folate synthesis. *Proc. Natl. Acad. Sci. U.S.A.* 101 13720–13725. 10.1073/pnas.0404208101 15365185 PMC518823

[B72] Diretto G. Al-Babili S. Tavazza R. Scossa F. Papacchioli V. Migliore M. (2010). Transcriptional-metabolic networks in beta-carotene-enriched potato tubers: the long and winding road to the Golden phenotype. *Plant Physiol.* 154 899–912. 10.1104/pp.110.159368 20671108 PMC2949014

[B73] Divi U. K. Krishna P. (2009). Brassinosteroid: a biotechnological target for enhancing crop yield and stress tolerance. *N. Biotechnol.* 26 131–136. 10.1016/j.nbt.2009.07.006 19631770

[B74] Domingos S. Fino J. Paulo O. S. Oliveira C. M. Goulao L. F. (2016). Molecular candidates for early-stage flower-to-fruit transition in stenospermocarpic table grape (*Vitis vinifera* L.) inflorescences ascribed by differential transcriptome and metabolome profiles. *Plant Sci.* 244 40–56. 10.1016/j.plantsci.2015.12.009 26810452

[B75] Domínguez T. Hernández M. L. Pennycooke J. C. Jiménez P. Martínez-Rivas J. M. Sanz C. (2010). Increasing omega-3 desaturase expression in tomato results in altered aroma profile and enhanced resistance to cold stress. *Plant Physiol.* 153 655–665. 10.1104/pp.110.154815 20382895 PMC2879794

[B76] Dong W. Cheng Z. J. Lei C. L. Wang X. L. Wang J. L. Wang J. (2014). Overexpression of folate biosynthesis genes in rice (*Oryza sativa* L.) and evaluation of their impact on seed folate content. *Plant Foods Hum. Nutr.* 69 379–385. 10.1007/s11130-014-0450-9 25432789

[B77] Dubos C. Stracke R. Grotewold E. Weisshaar B. Martin C. Lepiniec L. (2010). MYB transcription factors in *Arabidopsis*. *Trends Plant Sci.* 15 573–581. 10.1016/j.tplants.2010.06.005 20674465

[B78] Dudareva N. Pichersky E. (2008). Metabolic engineering of plant volatiles. *Curr. Opin. Biotechnol.* 19 181–189. 10.1016/j.copbio.2008.02.011 18394878

[B79] Dutta A. Shetty P. Bhat S. Ramachandra Y. Hegde S. (2012). A mass spectrometric study for comparative analysis and evaluation of metabolite recovery from plasma by various solvent systems. *J. Biomol. Tech.* 23 128–135. 10.7171/jbt.12-2304-001 23204928 PMC3437798

[B80] Dwivedi G. Jain S. Sharma M. P. (2011). Pongamia as a source of biodiesel in India. *Smart Grid Renew. Energy* 2 184–189. 10.4236/sgre.2011.23022

[B81] Edson C. Howell G. Flore J. (1995). Influence of crop load on photosynthesis and dry matter partitioning of seyval grapevines. III. Seasonal changes in dry matter partitioning, vine morphology, yield, and fruit composition. *Am. J. Enol. Vitic.* 46 478–485.

[B82] Elhiti M. Stasolla C. (2012). Abnormal development and altered hormone profile and sensitivity in *Arabidopsis* plants ectopically expressing *Brassica* shoot apical meristem genes. *J. Genet. Eng. Biotechnol.* 10 23–32. 10.1016/j.jgeb.2012.01.002

[B83] Espley R. V. Hellens R. P. Putterill J. Stevenson D. E. Kutty-Amma S. Allan A. C. (2007). Red colouration in apple fruit is due to the activity of the MYB transcription factor, MdMYB10. *Plant J.* 49 414–427. 10.1111/j.1365-313X.2006.02964.x 17181777 PMC1865000

[B84] Essigmann B. Güler S. Narang R. A. Linke D. Benning C. (1998). Phosphate availability affects the thylakoid lipid composition and the expression of SQD1 a gene required for sulfolipid biosynthesis in *Arabidopsis thaliana*. *Proc. Natl. Acad. Sci. U.S.A.* 95 1950–1955. 10.1073/pnas.95.4.1950 9465123 PMC19220

[B85] Fernández V. Guzmán-Delgado P. Graça J. Santos S. Gil L. (2016). Cuticle structure in relation to chemical composition: re-assessing the prevailing model. *Front. Plant Sci.* 7:427 10.3389/fpls.2016.00427 PMC4814898 27066059

[B86] Fernie A. R. (2003). Metabolome characterisation in plant system analysis. *Funct. Plant Biol.* 30 111–120. 10.1071/FP02163 32688998

[B87] Fernie A. R. Schauer N. (2009). Metabolomics-assisted breeding: a viable option for crop improvement? *Trends Genet.* 25 39–48. 10.1016/j.tig.2008.10.010 19027981

[B88] Fitzgerald G. J. Tausz M. O’Leary G. Mollah M. R. Tausz-Posch S. Seneweera S. (2016). Elevated atmospheric [CO_2_] can dramatically increase wheat yields in semi-arid environments and buffer against heat waves. *Glob. Change Biol.* 22 2269–2284. 10.1111/gcb.13263 26929390

[B89] Flamini R. De Rosso M. Bavaresco L. (2015). Study of grape polyphenols by liquid chromatography-high-resolution mass spectrometry (UHPLC/QTOF) and suspect screening analysis. *J. Anal. Methods Chem.* 2015:350259 10.1155/2015/350259 PMC4334975 25734021

[B90] Forkel M. Carvalhais N. Rödenbeck C. Keeling R. Heimann M. Thonicke K. (2016). Enhanced seasonal CO_2_ exchange caused by amplified plant productivity in northern ecosystems. *Science* 351 696–699. 10.1126/science.aac4971 26797146

[B91] Francki M. G. Hayton S. Gummer J. P. A. Rawlinson C. Trengove R. D. (2015). Metabolomic profiling and genomic analysis of wheat aneuploid lines to identify genes controlling biochemical pathways in mature grain. *Plant Biotechnol. J.* 14 649–660. 10.1111/pbi.12410 26032167 PMC11388812

[B92] Galpaz N. Wang Q. Menda N. Zamir D. Hirschberg J. (2008). Abscisic acid deficiency in the tomato mutant high-pigment 3 leading to increased plastid number and higher fruit lycopene content. *Plant J.* 53 717–730. 10.1111/j.1365-313X.2007.03362.x 17988221

[B93] Gilbert L. Alhagdow M. Nunes-Nesi A. Quemener B. Guillon F. Bouchet B. (2009). GDP-d-mannose 35-epimerase (GME) plays a key role at the intersection of ascorbate and non-cellulosic cell-wall biosynthesis in tomato. *Plant J.* 60 499–508. 10.1111/j.1365-313X.2009.03972.x 19619161

[B94] Gong L. Chen W. Gao Y. Liu X. Zhang H. Xu C. (2013). Genetic analysis of the metabolome exemplified using a rice population. *Proc. Natl. Acad. Sci. U.S.A.* 110 20320–20325. 10.1073/pnas.1319681110 24259710 PMC3864304

[B95] Good X. Kellogg J. A. Wagoner W. Langhoff D. Matsumura W. Bestwick R. K. (1994). Reduced ethylene synthesis by transgenic tomatoes expressing S-adenosylmethionine hydrolase. *Plant Mol. Biol.* 26 781–790. 10.1007/BF00028848 7999994

[B96] Graham P. H. Vance C. P. (2003). Legumes: importance and constraints to greater use. *Plant Physiol.* 131 872–877. 10.1104/pp.017004 12644639 PMC1540286

[B97] Groszmann M. Gonzalez-Bayon R. Lyons R. L. Greaves I. K. Kazan K. Peacock W. J. (2015). Hormone-regulated defense and stress response networks contribute to heterosis in *Arabidopsis* F1 hybrids. *Proc. Natl. Acad. Sci. U.S.A.* 112 E6397–E6406. 10.1073/pnas.1519926112 26527659 PMC4655576

[B98] Hall R. Beale M. Fiehn O. Hardy N. Sumner L. Bino R. (2002). Plant metabolomics: the missing link in functional genomics strategies. *Plant Cell* 14 1437–1440. 10.1105/tpc.140720 12119365 PMC543394

[B99] Hamerli D. Birch R. G. (2011). Transgenic expression of trehalulose synthase results in high concentrations of the sucrose isomer trehalulose in mature stems of field-grown sugarcane. *Plant Biotechnol. J.* 9 32–37. 10.1111/j.1467-7652.2010.00528.x 20492546

[B100] Hamilton A. J. Lycett G. W. Grierson D. (1990). Antisense gene that inhibits synthesis of the hormone ethylene in transgenic plants. *Nature* 346 284–287. 10.1038/346284a0

[B101] Hanson A. D. Roje S. (2001). One-carbon metabolism in higher plants. *Annu. Rev. Plant Physiol. Plant Mol. Biol.* 52 119–137. 10.1146/annurev.arplant.52.1.119 11337394

[B102] Härtel H. Dörmann P. Benning C. (2000). DGD1-independent biosynthesis of extraplastidic galactolipids after phosphate deprivation in *Arabidopsis*. *Proc. Natl. Acad. Sci. U.S.A.* 97 10649–10654. 10.1073/pnas.180320497 10973486 PMC27079

[B103] Hartwig T. Chuck G. S. Fujioka S. Klempien A. Weizbauer R. Potluri D. P. V. (2011). Brassinosteroid control of sex determination in maize. *Proc. Natl. Acad. Sci. U.S.A.* 108 19814–19819. 10.1073/pnas.1108359108 22106275 PMC3241820

[B104] Hatoum D. Annaratone C. Hertog M. L. A. T. M. Geeraerd A. H. Nicolai B. M. (2014). Targeted metabolomics study of “Braeburn” apples during long-term storage. *Postharvest Biol. Technol.* 96 33–41. 10.1016/j.postharvbio.2014.05.004

[B105] Hatoum D. Hertog M. L. A. T. M. Geeraerd A. H. Nicolai B. M. (2016). Effect of browning related pre- and post-harvest factors on the “Braeburn” apple metabolome during CA storage. *Postharvest Biol. Technol.* 111 106–116. 10.1016/j.postharvbio.2015.08.004

[B106] Heuvelink E. (1997). Effect of fruit load on dry matter partitioning in tomato. *Sci. Hortic.* 69 51–59. 10.1016/S0304-4238(96)00993-4

[B107] Heyman H. M. Dubery I. A. (2016). The potential of mass spectrometry imaging in plant metabolomics: a review. *Phytochem. Rev.* 15 297–316. 10.1016/j.jprot.2017.04.020

[B108] Hichri I. Barrieu F. Bogs J. Kappel C. Delrot S. Lauvergeat V. (2011). Recent advances in the transcriptional regulation of the flavonoid biosynthetic pathway. *J. Exp. Bot.* 62 2465–2483. 10.1093/jxb/erq442 21278228

[B109] Higashi Y. Okazaki Y. Myouga F. Shinozaki K. Saito K. (2015). Landscape of the lipidome and transcriptome under heat stress in *Arabidopsis thaliana*. *Sci. Rep.* 5:10533 10.1038/srep10533 PMC4444972 26013835

[B110] Hobbs D. H. Flintham J. E. Hills M. J. (2004). Genetic control of storage oil synthesis in seeds of Arabidopsis. *Plant Physiol.* 136 3341–3349. 10.1104/pp.104.049486 15466222 PMC523393

[B111] Högy P. Franzaring J. Schwadorf K. Breuer J. Schütze W. Fangmeier A. (2010a). Effects of free-air CO_2_ enrichment on energy traits and seed quality of oilseed rape. *Agric. Ecosyst. Environ.* 139 239–244. 10.1016/j.agee.2010.08.009

[B112] Högy P. Keck M. Niehaus K. Franzaring J. Fangmeier A. (2010b). Effects of atmospheric CO_2_ enrichment on biomass, yield and low molecular weight metabolites in wheat grain. *J. Cereal Sci.* 52 215–220. 10.1016/j.jcs.2010.05.009

[B113] Högy P. Wieser H. Köhler P. Schwadorf K. Breuer J. Franzaring J. (2009). Effects of elevated CO2 on grain yield and quality of wheat: Results from a 3-year free-air CO_2_ enrichment experiment. *Plant Biol.* 11 60–69. 10.1111/j.1438-8677.2009.00230.x 19778369

[B114] Hong J. Yang L. Zhang D. Shi J. (2016). Plant metabolomics: an indispensable system biology tool for plant science. *Int. J. Mol. Sci.* 17:E767 10.3390/ijms17060767 PMC4926328 27258266

[B115] Hou Q. Ufer G. Bartels D. (2016). Lipid signalling in plant responses to abiotic stress. *Plant Cell Environ.* 39 1029–1048. 10.1111/pce.12666 26510494

[B116] Hu C. Shi J. Quan S. Cui B. Kleessen S. Nikoloski Z. (2014). Metabolic variation between japonica and indica rice cultivars as revealed by non-targeted metabolomics. *Sci. Rep.* 4:5067 10.1038/srep05067 PMC5381408 24861081

[B117] Hu C. Tohge T. Chan S. A. Song Y. Rao J. Cui B. (2016). Identification of conserved and diverse metabolic shifts during rice grain development. *Sci. Rep.* 6:20942 10.1038/srep20942 PMC4748235 26860358

[B118] Idso S. B. Idso K. E. (2001). Effects of atmospheric CO_2_ enrichment on plant constituents related to animal and human health. *Exp. Bot.* 45 179–199. 10.1016/S0098-8472(00)00091-5 11275225

[B119] IPCC (2013). *Climate Change 2013: The Physical Science Basis.* Geneva: IPCC.

[B120] Issaq H. J. Van Q. N. Waybright T. J. Muschik G. M. Veenstra T. D. (2009). Analytical and statistical approaches to metabolomics research. *J. Sep. Sci.* 32 2183–2199. 10.1002/jssc.200900152 19569098

[B121] Jaakola L. (2013). New insights into the regulation of anthocyanin biosynthesis in fruits. *Trends Plant Sci.* 18 477–483. 10.1016/j.tplants.2013.06.003 23870661

[B122] Jang Y. K. Jung E. S. Lee H. A. Choi D. Lee C. H. (2015). Metabolomic characterization of hot pepper (*Capsicum annuum* “CM334”) during fruit development. *J. Agric. Food Chem.* 63 9452–9460. 10.1021/acs.jafc.5b03873 26465673

[B123] Jenks M. A. Joly R. A. Rich P. J. Axtell J. D. Ashworth E. N. (1994). Chemically induced cuticle mutation affecting epidermal conductance to water vapor and disease susceptibility in *Sorghum bicolor* (L.) Moench. *Plant Physiol.* 105 1239–1245. 10.1104/pp.105.4.1239 12232280 PMC159454

[B124] Ji X. Dong B. Shiran B. Talbot M. J. Edlington J. E. Hughes T. (2011). Control of abscisic acid catabolism and abscisic acid homeostasis is important for reproductive stage stress tolerance in cereals. *Plant Physiol.* 156 647–662. 10.1104/pp.111.176164 21502188 PMC3177265

[B125] Jia H. F. Chai Y. M. Li C. L. Lu D. Luo J. J. Qin L. (2011). Abscisic acid plays an important role in the regulation of strawberry fruit ripening. *Plant Physiol.* 157 188–199. 10.1104/pp.111.177311 21734113 PMC3165869

[B126] Johnson S. R. Lange B. M. (2015). Open-access metabolomics databases for natural product research: present capabilities and future potential. *Front. Bioeng. Biotechnol.* 3:22 10.3389/fbioe.2015.00022 PMC4349186 25789275

[B127] Jones R. A. Scott S. J. (1983). Improvement of tomato flavor by genetically increasing sugar and acid contents. *Euphytica* 32 845–855. 10.1007/BF00042166

[B128] Jorge T. F. Rodrigues J. A. Caldana C. Schmidt R. van Dongen J. T. Thomas-Oates J. (2016). Mass spectrometry-based plant metabolomics: metabolite responses to abiotic stress. *Mass Spectrom. Rev.* 35 620–649. 10.1002/mas.21449 25589422

[B129] Kaelin W. G. McKnight S. L. (2013). Influence of metabolism on epigenetics and disease. *Cell* 153 56–69. 10.1016/j.cell.2013.03.004 23540690 PMC3775362

[B130] Kage U. Karre S. Kushalappa A. C. Mccartney C. (2016). Identification and characterization of a fusarium head blight resistance gene *TaACT* in wheat QTL-2DL. *Plant Biotechnol. J.* 15 447–457. 10.1111/pbi.12641 27663684 PMC5362683

[B131] Kanai S. Ohkura K. Adu-Gyamfi J. J. Mohapatra P. K. Nguyen N. T. Saneoka H. (2007). Depression of sink activity precedes the inhibition of biomass production in tomato plants subjected to potassium deficiency stress. *J. Exp. Bot.* 58 2917–2928. 10.1093/jxb/erm149 17630289

[B132] Kang K. Kim Y.-S. Park S. Back K. (2009). Senescence-induced serotonin biosynthesis and its role in delaying senescence in rice leaves. *Plant Physiol.* 150 1380–1393. 10.1104/pp.109.138552 19439571 PMC2705024

[B133] Karlova R. Rosin F. M. Busscher-Lange J. Parapunova V. Do P. T. Fernie A. R. (2011). Transcriptome and metabolite profiling show that *APETALA2a* is a major regulator of tomato fruit ripening. *Plant Cell* 23 923–941. 10.1105/tpc.110.081273 21398570 PMC3082273

[B134] Kessler N. Walter F. Persicke M. Albaum S. P. Kalinowski J. Goesmann A. (2014). Allocator: an interactive web platform for the analysis of metabolomic LC-ESI-MS datasets, enabling semi-automated, user-revised compound annotation and mass isotopomer ratio analysis. *PLoS ONE* 9:e113909 10.1371/journal.pone.0113909 PMC4245236 25426929

[B135] Khakimov B. Jespersen B. Engelsen S. (2014). Comprehensive and comparative metabolomic profiling of wheat, barley, oat and rye using gas chromatography-mass spectrometry and advanced chemometrics. *Foods* 3 569–585. 10.3390/foods3040569 28234338 PMC5302243

[B136] Khush G. S. (2001). Green revolution: the way forward. *Nat. Rev. Genet.* 2 815–822. 10.1038/35093585 11584298

[B137] Klee H. J. Hayford M. B. Kretzmer K. A. Barry G. F. Kishore G. M. (1991). Control of ethylene synthesis by expression of a bacterial enzyme in transgenic tomato plants. *Plant Cell* 3 1187–1193. 10.1105/tpc.3.11.1187 1821764 PMC160085

[B138] Kliebenstein D. J. Gershenzon J. Mitchell-Olds T. (2001). Comparative quantitative trait loci mapping of aliphatic, indolic and benzylic glucosinolate production in *Arabidopsis thaliana* leaves and seeds. *Genetics* 159 359–370. 11560911 10.1093/genetics/159.1.359 PMC1461795

[B139] Komatsu S. Yamamoto A. Nakamura T. Nouri M. Z. Nanjo Y. Nishizawa K. (2011). Comprehensive analysis of mitochondria in roots and hypocotyls of soybean under flooding stress using proteomics and metabolomics techniques. *J. Proteome Res.* 10 3993–4004. 10.1021/pr2001918 21766870

[B140] Kosma D. K. Parsons E. P. Isaacson T. Lü S. Rose J. K. C. Jenks M. A. (2010). Fruit cuticle lipid composition during development in tomato ripening mutants. *Physiol. Plant* 139 107–117. 10.1111/j.1399-3054.2009.01342.x 20028482

[B141] Kumar N. Singh A. S. Kumari S. Reddy M. P. (2015). Biotechnological approaches for the genetic improvement of *Jatropha curcas* L.: a biodiesel plant. *Ind. Crops Prod.* 8 1172–1182. 10.1016/j.indcrop.2015.07.028

[B142] Kumar R. Khurana A. Sharma A. K. (2014). Role of plant hormones and their interplay in development and ripening of fleshy fruits. *J. Exp. Bot.* 65 4561–4575. 10.1093/jxb/eru277 25028558

[B143] Kumar Y. Zhang L. Panigrahi P. Dholakia B. B. Dewangan V. Chavan S. G. (2016). Fusarium oxysporum mediates systems metabolic reprogramming of chickpea roots as revealed by a combination of proteomics and metabolomics. *Plant Biotechnol J.* 14 1589–1603. 10.1111/pbi.12522 26801007 PMC5066658

[B144] Kunst L. Samuels L. (2009). Plant cuticles shine?: advances in wax biosynthesis and export. *Curr. Opin. Plant Biol.* 12 721–727. 10.1016/j.pbi.2009.09.009 19864175

[B145] Kusano M. Fukushima A. Kobayashi M. Hayashi N. Jonsson P. Moritz T. (2007). Application of a metabolomic method combining one-dimensional and two-dimensional gas chromatography-time-of-flight/mass spectrometry to metabolic phenotyping of natural variants in rice. *J. Chromatogr. B* 855 71–79. 10.1016/j.jchromb.2007.05.002 17556050

[B146] Kusano M. Saito K. (2012). Role of metabolomics in crop improvement. *J. Plant Biochem. Biotechnol.* 21 24–31. 10.1007/s13562-012-0131-4

[B147] Kusano M. Yang Z. Okazaki Y. Nakabayashi R. Fukushima A. Saito K. (2015). Using metabolomic approaches to explore chemical diversity in rice. *Mol. Plant.* 8 58–67. 10.1016/j.molp.2014.11.010 25578272

[B148] La Russa M. Bogen C. Uhmeyer A. Doebbe A. Filippone E. Kruse O. (2012). Functional analysis of three type-2 DGAT homologue genes for triacylglycerol production in the green microalga *Chlamydomonas reinhardtii*. *J. Biotechnol.* 162 13–20. 10.1016/j.jbiotec.2012.04.006 22542934

[B149] Lafont J. J. Espitia A. A. Sodré J. R. (2015). Potential vegetable sources for biodiesel production: cashew, coconut and cotton. *Mater. Renew. Sustain. Energy* 4:1 10.1007/s40243-014-0041-6

[B150] Langridge P. Fleury D. (2011). Making the most of ‘omics’ for crop breeding. *Trends Biotechnol.* 29 33–40. 10.1016/j.tibtech.2010.09.006 21030098

[B151] Lara I. Belgi B. Goulao L. F. (2015). A focus on the biosynthesis and composition of cuticle in fruits. *J. Agric. Food Chem.* 63 4005–4019. 10.1021/acs.jafc.5b00013 25850334

[B152] Larkindale J. Huang B. (2004). Thermotolerance and antioxidant systems in *Agrostis stolonifera*: involvement of salicylic acid, abscisic acid, calcium, hydrogen peroxide, and ethylene. *J. Plant Physiol.* 161 405–413. 10.1078/0176-1617-01239 15128028

[B153] Laurentin H. Ratzinger A. Karlovsky P. (2008). Relationship between metabolic and genomic diversity in sesame (*Sesamum indicum* L.). *BMC Genomics* 9:250 10.1186/1471-2164-9-250 PMC2440766 18510719

[B154] Lawlor D. W. Mitchell R. A. C. (1991). The effects of increasing CO_2_ on crop photosynthesis and productivity - a review of field studies. *Plant Cell Environ.* 14 807–818. 10.1111/j.1365-3040.1991.tb01444.x

[B155] Légeret B. Schulz-Raffelt M. Nguyen H. M. Auroy P. Beisson F. Peltier G. (2016). Lipidomic and transcriptomic analyses of *Chlamydomonas reinhardtii* under heat stress unveil a direct route for the conversion of membrane lipids into storage lipids. *Plant Cell Environ.* 39 834–847. 10.1111/pce.12656 26477535

[B156] Lei Z. Huhman D. V. Sumner L. W. (2011). Mass spectrometry strategies in metabolomics. *J. Biol. Chem.* 286 25435–25442. 10.1074/jbc.R111.238691 21632543 PMC3138266

[B157] Lewinsohn E. Schalechet F. Wilkinson J. Matsui K. Tadmor Y. Nam K. H. (2001). Enhanced levels of the aroma and flavor compound S-linalool by metabolic engineering of the terpenoid pathway in tomato fruits. *Plant Physiol.* 127 1256–1265. 10.1104/pp.010293 11706204 PMC129293

[B158] L’Haridon F. Besson-Bard A. Binda M. Serrano M. Abou-Mansour E. Balet F. (2011). A permeable cuticle is associated with the release of reactive oxygen species and induction of innate immunity. *PLoS Pathog.* 7:e1002148 10.1371/journal.ppat.1002148 PMC3145797 21829351

[B159] Li J. G. Li H. L. Peng S. Q. (2011). Three R2R3 MYB transcription factor genes from *Capsicum annuum* showing differential expression during fruit ripening. *Afr. J. Biotechnol.* 10 8267–8274. 10.5897/AJB10.2670

[B160] Li Q. Shen W. Zheng Q. Fowler D. B. Zou J. (2016). Adjustments of lipid pathways in plant adaptation to temperature stress. *Plant Signal. Behav.* 11:e1058461 10.1080/15592324.2015.1058461 PMC4871642 26734889

[B161] Li T. Heuvelink E. Marcelis L. F. M. (2015). Quantifying the source-sink balance and carbohydrate content in three tomato cultivars. *Front. Plant Sci.* 6:416 10.3389/fpls.2015.00416 PMC4456573 26097485

[B162] Lin-Wang K. Bolitho K. Grafton K. Kortstee A. Karunairetnam S. McGhie T. K. (2010). An R2R3 MYB transcription factor associated with regulation of the anthocyanin biosynthetic pathway in Rosaceae. *BMC Plant Biol.* 10:50 10.1186/1471-2229-10-50 PMC2923524 20302676

[B163] Lin-Wang K. McGhie T. K. Wang M. Liu Y. Warren B. Storey R. (2014). Engineering the anthocyanin regulatory complex of strawberry (*Fragaria vesca*). *Front. Plant Sci.* 5:651 10.3389/fpls.2014.00651 PMC4237049 25477896

[B164] Liu J. C. Temme A. A. Cornwell W. K. van Logtestijn R. S. P. Aerts R. Cornelissen J. H. C. (2016). Does plant size affect growth responses to water availability at glacial, modern and future CO_2_ concentrations? *Ecol. Res.* 31 213–227. 10.1007/s11284-015-1330-y

[B165] Liu L. Wei J. Zhang M. Zhang L. Li C. Wang Q. (2012). Ethylene independent induction of lycopene biosynthesis in tomato fruits by jasmonates. *J. Exp. Bot.* 63 5751–5762. 10.1093/jxb/ers224 22945939 PMC3467294

[B166] Liu Q. Xu J. Liu Y. Zhao X. Deng X. Guo L. (2007). A novel bud mutation that confers abnormal patterns of lycopene accumulation in sweet orange fruit (*Citrus sinensis* L. Osbeck). *J. Exp. Bot.* 58 4161–4171. 10.1093/jxb/erm273 18182424

[B167] Lodha T. D. Hembram P. Basak N. T. J. (2013). Proteomics: a successful approach to understand the molecular mechanism of plant-pathogen interaction. *Am. J. Plant Sci.* 4 1212–1226. 10.4236/ajps.2013.46149

[B168] Lohani S. Trivedi P. K. Nath P. (2004). Changes in activities of cell wall hydrolases during ethylene-induced ripening in banana: effect of 1-MCP, ABA and IAA. *Postharvest Biol. Technol.* 31 119–126. 10.1016/j.postharvbio.2003.08.001

[B169] Lommen A. van der Kamp H. J. Kools H. J. van der Lee M. K. van der Weg G. Mol H. G. (2012). metAlignID: a high-throughput software tool set for automated detection of trace level contaminants in comprehensive LECO two-dimensional gas chromatography time-of-flight mass spectrometry data. *J. Chromatogr. A* 1263 169–178. 10.1016/j.chroma.2012.09.056 23046623

[B170] Long M. Millar D. J. Kimura Y. Donovan G. Rees J. Fraser P. D., (2006). Metabolite profiling of carotenoid and phenolic pathways in mutant and transgenic lines of tomato: identification of a high antioxidant fruit line. *Phytochemistry* 67 1750–1757. 10.1016/j.phytochem.2006.02.022 16616263

[B171] Lopes A. S. Santa Cruz E. C. Sussulini A. Klassen A. (2017). “Metabolomic strategies involving mass spectrometry combined with liquid and gas chromatography,” in *Metabolomics: From Fundamentals to Clinical Applications*, ed. Sussulini A. (New York City, NY: Springer International Publishing), 77–98. 10.1007/978-3-319-47656-8_4 28132177

[B172] Ma P. Zhang Z. Zhou X. Yun Y. Liang Y. Lu H. (2016). Feature extraction from resolution perspective for gas chromatography-mass spectrometry datasets. *RSC Adv.* 6 113997–114004. 10.1039/C6RA17864B

[B173] Mach J. (2012). Identification of a novel maize protein important for paramutation at the purple plant1 locus. *Plant Cell* 24 1709–1709. 10.1105/tpc.112.240510

[B174] Makarevitch I. Thompson A. Muehlbauer G. J. Springer N. M. (2012). *Brd1* gene in maize encodes a brassinosteroid C-6 oxidase. *PLoS ONE* 7:e30798 10.1371/journal.pone.0030798 PMC3266906 22292043

[B175] Malathi B. Ramesh S. Venkateswara Rao K. Dashavantha Reddy V. (2006). *Agrobacterium*-mediated genetic transformation and production of semilooper resistant transgenic castor (*Ricinus communis* L.). *Euphytica* 147 441–449. 10.1007/s10681-005-9043-x

[B176] Manavalan L. P. Chen X. Clarke J. Salmeron J. Nguyen H. T. (2012). RNAi-mediated disruption of squalene synthase improves drought tolerance and yield in rice. *J. Exp. Bot.* 63 163–175. 10.1093/jxb/err258 21926092 PMC3245457

[B177] Manning K. Tör M. Poole M. Hong Y. Thompson A. J. King G. J. (2006). A naturally occurring epigenetic mutation in a gene encoding an SBP-box transcription factor inhibits tomato fruit ripening. *Nat. Genet.* 38 948–952. 10.1038/ng1841 16832354

[B178] Marcelis L. F. (1996). Sink strength as a determinant of dry matter partitioning in the whole plant. *J. Exp. Bot.* 47 1281–1291. 10.1093/jxb/47.Special_Issue.1281 21245260

[B179] Marti G. Erb M. Boccard J. Glauser G. Doyen G. R. Villard N. (2013). Metabolomics reveals herbivore-induced metabolites of resistance and susceptibility in maize leaves and roots. *Plant Cell Environ.* 36 621–639. 10.1111/pce.12002 22913585

[B180] Martin C. Butelli E. Petroni K. Tonelli C. (2011). How can research on plants contribute to promoting human health? *Plant Cell* 23 1685–1699. 10.1105/tpc.111.083279 21586682 PMC3123949

[B181] Mathews H. Clendennen S. K. Caldwell C. G. Liu X. L. Connors K. Matheis N. (2003). Activation tagging in tomato identifies a transcriptional regulator of anthocyanin biosynthesis, modification, and transport. *Plant Cell* 15 1689–1703. 10.1105/tpc.012963 12897245 PMC167162

[B182] Matsuda F. Hirai M. Y. Sasaki E. Akiyama K. Yonekura-Sakakibara K. Provart N. J. (2010). AtMetExpress development: a phytochemical atlas of Arabidopsis development. *Plant Physiol.* 152 566–578. 10.1104/pp.109.148031 20023150 PMC2815869

[B183] Matsuda F. Okazaki Y. Oikawa A. Kusano M. Nakabayashi R. Kikuchi J. (2012). Dissection of genotype-phenotype associations in rice grains using metabolome quantitative trait loci analysis. *Plant J.* 70 624–636. 10.1111/j.1365-313X.2012.04903.x 22229385

[B184] Mazurek S. Garroum I. Daraspe J. De Bellis D. Olsson V. Mucciolo A. (2017). Connecting cutin composition to cuticle ultrastructure and physical properties of Arabidopsis petals. *Plant Physiol.* 173 1146–1163. 10.1104/pp.16.01637 27994007 PMC5291042

[B185] Mazzucotelli E. Tartari A. Cattivelli L. Forlani G. (2006). Metabolism of γ-aminobutyric acid during cold acclimation and freezing and its relationship to frost tolerance in barley and wheat. *J. Exp. Bot.* 57 3755–3766. 10.1093/jxb/erl141 16997899

[B186] Medina-Puche L. Cumplido-Laso G. Amil-Ruiz F. Hoffmann T. Ring L. Rodríguez-Franco A. (2014). MYB10 plays a major role in the regulation of flavonoid/phenylpropanoid metabolism during ripening of *Fragaria × ananassa* fruits. *J. Exp. Bot.* 65 401–417. 10.1093/jxb/ert377 24277278

[B187] Menu T. Saglio P. Granot D. Dai N. Raymond P. Ricard B. (2004). High hexokinase activity in tomato fruit perturbs carbon and energy metabolism and reduces fruit and seed size. *Plant Cell Environ.* 27 89–98. 10.1046/j.0016-8025.2003.01128.x

[B188] Miglietta F. Magliulo V. Bindi M. Cerio L. Vaccari F. P. Loduca V. (1998). Free air CO2 enrichment of potato (*Solanum tuberosum* L.): development, growth and yield. *Glob. Chang Biol.* 4 163–172. 10.1046/j.1365-2486.1998.00120.x

[B189] Mikulic-Petkovsek M. Schmitzer V. Slatnar A. Weber N. Veberic R. Stampar F. (2013). Alteration of the content of primary and secondary metabolites in strawberry fruit by *Colletotrichum nymphaeae* infection. *J. Agric. Food Chem.* 61 5987–5995. 10.1021/jf402105g 23734881

[B190] Misra B. B. van der Hooft J. J. (2016). Updates in metabolomics tools and resources: 2014–2015. *Electrophoresis* 37 86–110. 10.1002/elps.201500417 26464019

[B191] Moco S. Forshed J. De Vos R. C. H. Bino R. J. Vervoort J. (2008). Intra- and inter-metabolite correlation spectroscopy of tomato metabolomics data obtained by liquid chromatography-mass spectrometry and nuclear magnetic resonance. *Metabolomics* 4 202–215. 10.1007/s11306-008-0112-8

[B192] Morgan P. B. Bollero G. A. Nelson R. L. Dohleman F. G. Long S. P. (2005). Smaller than predicted increase in aboveground net primary production and yield of field-grown soybean under fully open-air [CO2] elevation. *Glob. Chang Biol.* 11 1856–1865. 10.1111/j.1365-2486.2005.001017.x

[B193] Morreel K. Goeminne G. Storme V. Sterck L. Ralph J. Coppieters W. (2006). Genetical metabolomics of flavonoid biosynthesis in Populus: a case study. *Plant J.* 47 224–237. 10.1111/j.1365-313X.2006.02786.x 16774647

[B194] Moulis J. M. (2010). Cellular mechanisms of cadmium toxicity related to the homeostasis of essential metals. *Biometals* 23 877–896. 10.1007/s10534-010-9336-y 20524046

[B195] Muscolo A. Junker A. Klukas C. Weigelt-Fischer K. Riewe D. Altmann T. (2015). Phenotypic and metabolic responses to drought and salinity of four contrasting lentil accessions. *J. Exp. Bot.* 66 5467–5480. 10.1093/jxb/erv208 25969553 PMC4585415

[B196] Nagpala E. G. Guidarelli M. Gasperotti M. Masuero D. Bertolini P. Vrhovsek U. (2016). Polyphenols variation in fruits of the susceptible strawberry cultivar alba during ripening and upon fungal pathogen interaction and possible involvement in unripe fruit tolerance. *J. Agric. Food Chem.* 64 1869–1878. 10.1021/acs.jafc.5b06005 26895094

[B197] Naqvi S. Zhu C. Farre G. Ramessar K. Bassie L. Breitenbach J. (2009). Transgenic multivitamin corn through biofortification of endosperm with three vitamins representing three distinct metabolic pathways. *Proc. Natl. Acad. Sci. U.S.A.* 106 7762–7767. 10.1073/pnas.0901412106 19416835 PMC2683132

[B198] Narayanan S. Prasad P. V. V. Welti R. (2016a). Wheat leaf lipids during heat stress: II. Lipids experiencing coordinated metabolism are detected by analysis of lipid co-occurrence. *Plant Cell Environ.* 39 608–617. 10.1111/pce.12648 26436445 PMC5141584

[B199] Narayanan S. Tamura P. J. Roth M. R. Prasad P. V. Welti R. (2016b). Wheat leaf lipids during heat stress: I. High day and night temperatures result in major lipid alterations. *Plant Cell Environ.* 39 787–803. 10.1111/pce.12649 26436679 PMC5102054

[B200] Nardozza S. Boldingh H. L. Osorio S. Höhne M. Wohlers M. Gleave A. P. (2013). Metabolic analysis of kiwifruit (*Actinidia deliciosa*) berries from extreme genotypes reveals hallmarks for fruit starch metabolism. *J. Exp. Bot.* 64 5049–5063. 10.1093/jxb/ert293 24058160 PMC3830485

[B201] Natera S. H. Hill C. B. Rupasinghe T. W. Roessner U. (2016). Salt-stress induced alterations in the root lipidome of two barley genotypes with contrasting responses to salinity. *Funct. Plant Biol.* 43 207–219. 10.1093/jxb/erp198 32480454

[B202] Nguyen C. V. Vrebalov J. T. Gapper N. E. Zheng Y. Zhong S. Fei Z. (2014). Tomato GOLDEN2-LIKE transcription factors reveal molecular gradients that function during fruit development and ripening. *Plant Cell* 26 585–601. 10.1105/tpc.113.118794 24510723 PMC3967027

[B203] Niu S. S. Xu C. J. Zhang W. S. Zhang B. Li X. Lin-Wang K. (2010). Coordinated regulation of anthocyanin biosynthesis in Chinese bayberry (*Myrica rubra*) fruit by a R2R3 MYB transcription factor. *Planta* 231 887–899. 10.1007/s00425-009-1095-z 20183921

[B204] Nole-Wilson S. Rueschhoff E. E. Bhatti H. Franks R. G. (2010). Synergistic disruptions in seuss cyp85A2 double mutants reveal a role for brassinolide synthesis during gynoecium and ovule development. *BMC Plant Biol.* 10:198 10.1186/1471-2229-10-198 PMC2956547 20836864

[B205] Nunes A. C. S. Kalkmann D. C. Aragão F. J. L. (2009). Folate biofortification of lettuce by expression of a codon optimized chicken GTP cyclohydrolase I gene. *Transgenic Res.* 18 661–667. 10.1007/s11248-009-9256-1 19322672

[B206] Nunes-Nesi A. Carrari F. Lytovchenko A. Smith A. M. Loureiro M. E. Ratcliffe R. G. (2005). Enhanced photosynthetic performance and growth as a consequence of decreasing mitochondrial malate dehydrogenase activity in transgenic tomato plants. *Plant Physiol.* 137 611–622. 10.1104/pp.104.055566 15665243 PMC1065362

[B207] Oa N. M. W. Martel C. Vrebalov J. Tafelmeyer P. Giovannoni J. J. (2011). The tomato MADS-box transcription factor RIPENING INHIBITOR interacts with promoters involved in numerous ripening processes in a COLORLESS NONRIPENING-dependent manner. *Plant Physiol.* 157 1568–1579. 10.1104/pp.111.181107 21941001 PMC3252172

[B208] Obata T. Witt S. Lisec J. Palacios-Rojas N. Florez-Sarasa I. Yousfi S. (2015). Metabolite profiles of maize leaves in drought, heat and combined stress field trials reveal the relationship between metabolism and grain yield. *Plant Physiol.* 169 2665–2683. 10.1104/pp.15.01164 26424159 PMC4677906

[B209] Oeller P. W. Lu M. W. Taylor L. P. Pike D. A. Theologis A. (1991). Reversible inhibition of tomato fruit senescence by antisense RNA. *Science* 254 437–439. 10.1126/science.1925603 1925603

[B210] Ogbaga C. C. Stepien P. Dyson B. C. Rattray N. J. Ellis D. I. Goodacre R. (2016). Biochemical analyses of sorghum varieties reveal differential responses to drought. *PLoS ONE* 11:e0154423 10.1371/journal.pone.0154423 PMC4859509 27153323

[B211] Oikawa A. Matsuda F. Kusano M. Okazaki Y. Saito K. (2008). Rice metabolomics. *Rice* 1 63–71. 10.1007/s12284-008-9009-4

[B212] Oikawa A. Otsuka T. Nakabayashi R. Jikumaru Y. Isuzugawa K. Murayama H. (2015). Metabolic profiling of developing pear fruits reveals dynamic variation in primary and secondary metabolites, including plant hormones. *PLoS ONE* 10:e0131408 10.1371/journal.pone.0131408 PMC4500446 26168247

[B213] Okazaki Y. Saito K. (2016). Integrated metabolomics and phytochemical genomics approaches for studies on rice. *Gigascience* 5 11 10.1186/s13742-016-0116-7 PMC4774183 26937280

[B214] Ortiz-Martínez V. M. Salar-García M. J. Palacios-Nereo F. J. Olivares-Carrillo P. Quesada-Medina J. De los Ríos A. P. (2016). In-depth study of the transesterification reaction of *Pongamia pinnata* oil for biodiesel production using catalyst-free supercritical methanol process. *J. Supercrit. Fluids* 113 23–30. 10.1016/j.supflu.2016.03.009

[B215] Osanai Y. Tissue D. T. Bange M. P. Anderson I. C. Braunack M. V. Singh B. K. (2017). Plant-soil interactions and nutrient availability determine the impact of elevated CO2 and temperature on cotton productivity. *Plant Soil* 410 87–102. 10.1007/s11104-016-2981-3

[B216] Osorio S. Alba R. Damasceno C. M. Lopez-Casado G. Lohse M. Zanor M. I. (2011). Systems biology of tomato fruit development: combined transcript, protein, and metabolite analysis of tomato transcription factor (nor, rin) and ethylene receptor (Nr) mutants reveals novel regulatory interactions. *Plant Physiol.* 157 405–425. 10.1104/pp.111.175463 21795583 PMC3165888

[B217] Osorio S. Ruan Y.-L. Fernie A. R. (2014). An update on source-to-sink carbon partitioning in tomato. *Front. Plant Sci.* 5:516 10.3389/fpls.2014.00516 PMC4186278 25339963

[B218] Paine J. A. Shipton C. A. Chaggar S. Howells R. M. Kennedy M. J. Vernon G. (2005). Improving the nutritional value of Golden Rice through increased pro-vitamin A content. *Nat. Biotechnol.* 23 482–487. 10.1038/nbt1082 15793573

[B219] Pal M. Chaturvedi A. K. Pandey S. K. Bahuguna R. N. Khetarpal S. Anand A. (2014). Rising atmospheric CO2 may affect oil quality and seed yield of sunflower (*Helianthus annuus* L.). *Acta Physiol. Plant.* 36 2853–2861. 10.1007/s11738-014-1651-4

[B220] Pan Z. Li Y. Deng X. Xiao S. (2014). Non-targeted metabolomic analysis of orange (*Citrus sinensis* [L.] Osbeck) wild type and bud mutant fruits by direct analysis in real-time and HPLC-electrospray mass spectrometry. *Metabolomics* 10 508–523. 10.1007/s11306-013-0597-7

[B221] Pandey M. K. Roorkiwal M. Singh V. K. Ramalingam A. Kudapa H. Thudi M. (2016). Emerging genomic tools for legume breeding: current status and future perspectives. *Front. Plant Sci.* 7:455 10.3389/fpls.2016.00455 PMC4852475 27199998

[B222] Parent B. Hachez C. Redondo E. Simonneau T. Chaumont F. Tardieu F. (2009). Drought and abscisic acid effects on aquaporin content translate into changes in hydraulic conductivity and leaf growth rate: a trans-scale approach. *Plant Physiol.* 149 2000–2012. 10.1104/pp.108.130682 19211703 PMC2663730

[B223] Parry M. A. Hawkesford M. J. (2012). An integrated approach to crop genetic improvement. *J. Integr. Plant Biol.* 54 250–259. 10.1111/j.1744-7909.2012.01109.x 22348899

[B224] Paul B. Barnes S. Demark-Wahnefried W. Morrow C. Salvador C. Skibola C. (2015). Influences of diet and the gut microbiome on epigenetic modulation in cancer and other diseases. *Clin. Epigenet.* 7 112 10.1186/s13148-015-0144-7 PMC4609101 26478753

[B225] Pegasus H. T. (2007). *ChromaTOF Software Instruction Manual, Version 3.3.* Available at: http://www.scielo.br/scielo.php?script=sci_nlinks\&ref=000133\&pid=S0100-4042201100060000900032\&lng=en

[B226] Perez-Fons L. Wells T. Corol D. I. Ward J. L. Gerrish C. Beale M. H. (2014). A genome-wide metabolomic resource for tomato fruit from *Solanum pennellii*. *Sci. Rep.* 4:3859 10.1038/srep03859 PMC3900926 24457419

[B227] Petersen A. K. Zeilinger S. Kastenmüller G. Römisch-Margl W. Brugger M. Peters A. (2014). Epigenetics meets metabolomics: an epigenome-wide association study with blood serum metabolic traits. *Hum. Mol. Genet.* 23 534–545. 10.1093/hmg/ddt430 24014485 PMC3869358

[B228] Picton S. Barton S. L. Bouzayen M. Hamilton A. J. Grierson D. (1993). Altered fruit ripening and leaf senescence in tomatoes expressing an antisense ethylene-forming enzyme transgene. *Plant J.* 3 469–481. 10.1111/j.1365-313X.1993.tb00167.x

[B229] Pilu R. Panzeri D. Cassani E. Cerino Badone F. Landoni M. Nielsen E. (2009). A paramutation phenomenon is involved in the genetics of maize low phytic acid1-241 (lpa1-241) *trait*. *Heredity (Edinb).* 102 236–245. 10.1038/hdy.2008.96 18781168

[B230] Pinheiro C. Passarinho J. A. Ricardo C. P. (2004). Effect of drought and rewatering on the metabolism of *Lupinus albus* organs. *J. Plant Physiol.* 161 1203–1210. 10.1016/j.jplph.2004.01.016 15602812

[B231] Prasad M. N. V. (1995). Cadmium toxicity and tolerance in vascular plants. *Environ. Exp. Bot.* 35 525–545. 10.1016/0098-8472(95)00024-0

[B232] Qin H. Gu Q. Zhang J. Sun L. Kuppu S. Zhang Y. (2011). Regulated expression of an isopentenyltransferase gene (IPT) in peanut significantly improves drought tolerance and increases yield under field conditions. *Plant Cell Physiol.* 52 1904–1914. 10.1093/pcp/pcr125 21920877

[B233] Qu J. Mao H. Chen W. Gao S. Bai Y. Sun Y. (2012). Development of marker-free transgenic Jatropha plants with increased levels of seed oleic acid. *Biotechnol. Biofuels* 5:10 10.1186/1754-6834-5-10 PMC3316142 22377043

[B234] Quadrana L. Almeida J. Asís R. Duffy T. Dominguez P. G. Bermúdez L. (2014). Natural occurring epialleles determine vitamin E accumulation in tomato fruits. *Nat. Commun.* 5 3027 10.1038/ncomms5027 24967512

[B235] Raffaele S. Leger A. Roby D. (2009). Very long chain fatty acid and lipid signaling in the response of plants to pathogens. *Plant Signal. Behav.* 4 94–99. 10.4161/psb.4.2.7580 19649180 PMC2637489

[B236] Rajapakse N. C. He C. Cisneros-Zevallos L. Davies F. T. (2009). Hypobaria and hypoxia affects growth and phytochemical contents of lettuce. *Sci. Hortic.* 122 171–178. 10.1016/j.scienta.2009.05.002

[B237] Ralston-Hooper K. Hopf A. Oh C. Zhang X. Adamec J. Sepúlveda M. S. (2008). Development of GCxGC/TOF-MS metabolomics for use in ecotoxicological studies with invertebrates. *Aquat. Toxicol.* 88 48–52. 10.1016/j.aquatox.2008.03.002 18423646

[B238] Ramalingam A. Kudapa H. Pazhamala L. T. Weckwerth W. Varshney R. K. (2015). Proteomics and metabolomics: two emerging areas for legume improvement. *Front. Plant Sci.* 6:1116 10.3389/fpls.2015.01116 PMC4689856 26734026

[B239] Ramautar R. de Jong G. J. (2014). Recent developments in liquid-phase separation techniques for metabolomics. *Bioanalysis* 6 1011–1026. 10.4155/bio.14.51 24806908

[B240] Redestig H. Kusano M. Ebana K. Kobayashi M. Oikawa A. Okazaki Y. (2011). Exploring molecular backgrounds of quality traits in rice by predictive models based on high coverage metabolomics. *BMC Syst. Biol.* 5:176 10.1186/1752-0509-5-176 PMC3305925 22034874

[B241] Reich M. van den Meerakker A. N. Parmar S. Hawkesford M. J. De Kok L. J. (2016). Temperature determines size and direction of effects of elevated CO2 and nitrogen form on yield quantity and quality of Chinese cabbage. *Plant Biol.* 18 63–75. 10.1111/plb.12396 26390257

[B242] Reich P. B. Hungate B. A. Luo Y. (2006). Carbon-nitrogen interactions in terrestrial ecosystems in response to rising atmospheric carbon dioxide. *Annu. Rev. Ecol. Evol. Syst.* 37 611–636. 10.1146/annurev.ecolsys.37.091305.110039

[B243] Reina-Pinto J. J. Yephremov A. (2009). Surface lipids and plant defenses. *Plant Physiol. Biochem.* 47 540–549. 10.1016/j.plaphy.2009.01.004 19230697

[B244] Ricroch A. E. Berge J. B. Kuntz M. (2011). Evaluation of genetically engineered crops using transcriptomic, proteomic, and metabolomic profiling techniques. *Plant Physiol.* 155 1752–1761. 10.1104/pp.111.173609 21350035 PMC3091128

[B245] Riedelsheimer C. Czedik-Eysenberg A. Grieder C. Lisec J. Technow F. Sulpice R. (2012). Genomic and metabolic prediction of complex heterotic traits in hybrid maize. *Nat. Genet.* 44 217–220. 10.1038/ng.1033 22246502

[B246] Rocha M. Licausi F. Araujo W. L. Nunes-Nesi A. Sodek L. Fernie A. R. (2010a). Glycolysis and the tricarboxylic acid cycle are linked by alanine aminotransferase during hypoxia induced by waterlogging of *Lotus japonicus*. *Plant Physiol.* 152 1501–1513. 10.1104/pp.109.150045 20089769 PMC2832266

[B247] Rocha M. Sodek L. Licausi F. Hameed M. W. Dornelas M. C. van Dongen J. T. (2010b). Analysis of alanine aminotransferase in various organs of soybean (*Glycine max*) and in dependence of different nitrogen fertilisers during hypoxic stress. *Amino Acids* 39 1043–1053. 10.1007/s00726-010-0596-1 20414691 PMC2945468

[B248] Rochfort S. J. Trenerry V. C. Imsic M. Panozzo J. Jones R. (2008). Class targeted metabolomics: ESI ion trap screening methods for glucosinolates based on MSn fragmentation. *Phytochemistry* 69 1671–1679. 10.1016/j.phytochem.2008.02.010 18396302

[B249] Rogers A. Gibon Y. Stitt M. Morgan P. B. Bernacchi C. J. Ort D. R. (2006). Increased C availability at elevated carbon dioxide concentration improves N assimilation in a legume. *Plant Cell Environ.* 29 1651–1658. 10.1111/j.1365-3040.2006.01549.x 16898025

[B250] Rowe H. C. Hansen B. G. Halkier B. A. Kliebenstein D. J. (2008). Biochemical networks and epistasis shape the *Arabidopsis thaliana* metabolome. *Plant Cell* 20 1199–1216. 10.1105/tpc.108.058131 18515501 PMC2438456

[B251] Sakamoto H. Maruyama K. Sakuma Y. Meshi T. Iwabuchi M. Shinozaki K. (2004). Arabidopsis Cys2/His2-type zinc-finger proteins function as transcription repressors under drought, cold, and high-salinity stress conditions. *Plant Physiol.* 136 2734–2746. 10.1104/pp.104.046599 15333755 PMC523337

[B252] Sakamoto T. Matsuoka M. (2008). Identifying and exploiting grain yield genes in rice. *Curr. Opin. Plant Biol.* 11 209–214. 10.1016/j.pbi.2008.01.009 18343712

[B253] Saladié M. Matas A. J. Isaacson T. Jenks M. A. Goodwin S. M. Niklas K. J. (2007). A reevaluation of the key factors that influence tomato fruit softening and integrity. *Plant Physiol.* 144 1012–1028. 10.1104/pp.107.097477 17449643 PMC1914194

[B254] Salekdeh G. H. Komatsu S. (2007). Crop proteomics: aim at sustainable agriculture of tomorrow. *Proteomics* 7 2976–2996. 10.1002/pmic.200700181 17639607

[B255] Samuels L. Kunst L. Jetter R. (2008). Sealing plant surfaces?: cuticular wax formation by epidermal cells. *Annu. Rev. Plant Biol.* 59 683–707. 10.1146/annurev.arplant.59.103006.093219 18251711

[B256] Sanchez D. H. Pieckenstain F. L. Escaray F. Erban A. Kraemer U. Udvardi M. K. (2011). Comparative ionomics and metabolomics in extremophile and glycophytic Lotus species under salt stress challenge the metabolic pre-adaptation hypothesis. *Plant Cell Environ.* 34 605–617. 10.1111/j.1365-3040.2010.02266.x 21251019

[B257] Sarwar M. Sarwar M. Sarwar M. Qadri N. Moghal S. (2013). The importance of cereals (Poaceae: Gramineae) nutrition in human health: a review. *J. Cereal Oilseeds* 4 32–35. 10.1016/j.plantsci.2015.08.023

[B258] Saydut A. Erdogan S. Kafadar A. B. Kaya C. Aydin F. Hamamci C. (2016). Process optimization for production of biodiesel from hazelnut oil, sunflower oil and their hybrid feedstock. *Fuel* 183 512–517. 10.1016/j.fuel.2016.06.114

[B259] Schauer N. Semel Y. Roessner U. Gur A. Balbo I. Carrari F. (2006). Comprehensive metabolic profiling and phenotyping of interspecific introgression lines for tomato improvement. *Nat. Biotechnol.* 24 447–454. 10.1038/nbt1192 16531992

[B260] Schauer N. Zamir D. Fernie A. R. (2005). Metabolic profiling of leaves and fruit of wild species tomato: a survey of the *Solanum lycopersicum* complex. *J. Exp. Bot.* 56 297–307. 10.1093/jxb/eri057 15596477

[B261] Schippers P. Lurling M. Scheffer M. (2004). Increase of atmospheric CO2 promotes phytoplankton productivity. *Ecol. Lett.* 7 446–451. 10.1111/j.1461-0248.2004.00597.x

[B262] Schmid I. Franzaring J. Müller M. Brohon N. Calvo O. C. Högy P. (2016). Effects of CO2 enrichment and drought on photosynthesis, growth and yield of an old and a modern barley cultivar. *J. Agron. Crop Sci.* 202 81–95. 10.1111/jac.12127

[B263] Schrader W. Klein H. W. (2004). Liquid chromatography/Fourier transform ion cyclotron resonance mass spectrometry (LC-FTICR MS): an early overview. *Anal. Bioanal. Chem.* 379 1013–1024. 10.1007/s00216-004-2675-1 15221183

[B264] Schreiber G. Reuveni M. Evenor D. Oren-Shamir M. Ovadia R. Sapir-Mir M. (2012). ANTHOCYANIN1 from *Solanum chilense* is more efficient in accumulating anthocyanin metabolites than its *Solanum lycopersicum* counterpart in association with the ANTHOCYANIN FRUIT phenotype of tomato. *Theor. Appl. Genet.* 124 295–307. 10.1007/s00122-011-1705-6 21947299

[B265] Seo D. J. Patrick C. J. Kennealy P. J. (2008). Role of serotonin and dopamine system interactions in the neurobioloy of impulsive aggression and its comorbidity with other clinical disorders. *Aggress. Violent Behav.* 13 383–395. 10.1016/j.avb.2008.06.003 19802333 PMC2612120

[B266] Serrano M. Coluccia F. Torres M. L’Haridon F. Métraux J. P. (2014). The cuticle and plant defense to pathogens. *Front. Plant Sci.* 5:274 10.3389/fpls.2014.00274 PMC4056637 24982666

[B267] Setha S. (2012). Roles of abscisic acid in fruit ripening. *Walailak J. Sci. Technol.* 9 297–308.

[B268] Shah J. (2005). Lipids, lipases, and lipid-modifying enzymes in plant disease resistance. *Annu. Rev. Phytopathol.* 43 229–260. 10.1146/annurev.phyto.43.040204.135951 16078884

[B269] Shelden M. C. Dias D. A. Jayasinghe N. S. Bacic A. Roessner U. (2016). Root spatial metabolite profiling of two genotypes of barley (*Hordeum vulgare* L.) reveals differences in response to short-term salt stress. *J. Exp. Bot.* 67 3731–3745. 10.1093/jxb/erw059 26946124 PMC4896359

[B270] Sheng L. Shen D. Yang W. Zhang M. Zeng Y. Xu J. (2017). GABA pathway rate-limit citrate degradation in postharvest citrus fruit evidence from HB Pumelo (*Citrus grandis*)× Fairchild (*Citrus reticulata*) hybrid population. *J. Agric. Food Chem.* 65 1669–1676. 10.1021/acs.jafc.6b05237 28150945

[B271] Silvente S. Sobolev A. P. Lara M. (2012). Metabolite adjustments in drought tolerant and sensitive soybean genotypes in response to water stress. *PLoS ONE* 7:e38554 10.1371/journal.pone.0038554 PMC3369847 22685583

[B272] Simó C. Ibáñez C. Valdés A. Cifuentes A. García-Cañas V. (2014). Metabolomics of genetically modified crops. *Int. J. Mol. Sci.* 15 18941–18966. 10.3390/ijms151018941 25334064 PMC4227254

[B273] Slisz A. M. Breksa A. P. Mishchuk D. O. McCollum G. Slupsky C. M. (2012). Metabolomic analysis of citrus infection by “Candidatus Liberibacter” reveals insight into pathogenicity. *J. Proteome Res.* 11 4223–4230. 10.1021/pr300350x 22698301

[B274] Smith S. D. Huxman T. E. Zitzer S. F. Charlet T. N. Housman D. C. Coleman J. S. (2000). Elevated CO2 increases productivity and invasive species success in an arid ecosystem. *Nature* 408 79–82. 10.1038/35040544 11081510

[B275] Son H. S. Hwang G. S. Ahn H. J. Park W. M. Lee C. H. Hong Y. S. (2009). Characterization of wines from grape varieties through multivariate statistical analysis of 1H NMR spectroscopic data. *Food Res. Int.* 42 1483–1491. 10.1016/j.foodres.2009.08.006

[B276] Song Q. Zhang T. Stelly D. M. Chen Z. J. (2017). Epigenomic and functional analyses reveal roles of epialleles in the loss of photoperiod sensitivity during domestication of allotetraploid cottons. *Genome Biol.* 18:99 10.1186/s13059-017-1229-8 PMC5450403 28558752

[B277] Spencer J. P. E. Kuhnle G. G. C. Hajirezaei M. Mock H.-P. Sonnewald U. Rice-Evans C. (2005). The genotypic variation of the antioxidant potential of different tomato varieties. *Free Radic. Res.* 39 1005–1016. 10.1080/10715760400022293 16087482

[B278] Spicher L. Glauser G. Kessler F. (2016). Lipid antioxidant and galactolipid remodeling under temperature stress in tomato plants. *Front. Plant Sci.* 7:167 10.3389/fpls.2016.00167 PMC4756161 26925083

[B279] Spollen W. G. LeNoble M. E. Samuels T. D. Bernstein N. Sharp R. E. (2000). Abscisic acid accumulation maintains maize primary root elongation at low water potentials by restricting ethylene production. *Plant Physiol.* 122 967–976. 10.1104/pp.122.3.967 10712561 PMC58933

[B280] Srivastava M. Dwivedi U. (2000). Delayed ripening of banana fruit by salicylic acid. *Plant Sci.* 158 87–96. 10.1016/S0168-9452(00)00304-6 10996248

[B281] Storozhenko S. De Brouwer V. Volckaert M. Navarrete O. Blancquaert D. Zhang G. F. (2007). Folate fortification of rice by metabolic engineering. *Nat. Biotechnol.* 25 1277–1279. 10.1038/nbt1351 17934451

[B282] Strauch R. C. Svedin E. Dilkes B. Chapple C. Li X. (2015). Discovery of a novel amino acid racemase through exploration of natural variation in *Arabidopsis thaliana*. *Proc. Natl. Acad. Sci. U.S.A.* 112 11726–11731. 10.1073/pnas.1503272112 26324904 PMC4577208

[B283] Sujatha M. Reddy T. P. Mahasi M. J. (2008). Role of biotechnological interventions in the improvement of castor (*Ricinus communis* L.) and *Jatropha curcas* L. *Biotechnol. Adv.* 26 424–435. 10.1016/j.biotechadv.2008.05.004 18579331

[B284] Sun L. Sun Y. Zhang M. Wang L. Ren J. Cui M. (2012a). Suppression of 9-cis-epoxycarotenoid dioxygenase, which encodes a key enzyme in abscisic acid biosynthesis, alters fruit texture in transgenic tomato. *Plant Physiol.* 158 283–298. 10.1104/pp.111.186866 22108525 PMC3252109

[B285] Sun P. Mantri N. Lou H. Hu Y. Sun D. Zhu Y. (2012). Effects of elevated CO2 and temperature on yield and fruit quality of strawberry (*Fragaria* x *ananassa* Duch.) at two levels of nitrogen application. *PLoS ONE* 7:e41000 10.1371/journal.pone.0041000 PMC3404062 22911728

[B286] Suravajhala P. Kogelman L. J. A. Kadarmideen H. N. (2016). Multi-omic data integration and analysis using systems genomics approaches: methods and applications in animal production, health and welfare. *Genet. Sel. Evol.* 48:38 10.1186/s12711-016-0217-x PMC4850674 27130220

[B287] Sutka J. Snape J. W. (1989). Location of a gene for frost resistance on chromosome 5A of wheat. *Euphytica* 42 41–44. 10.1270/jsbbs.63.58

[B288] Szymanski J. Brotman Y. Willmitzer L. Cuadros-Inostroza Á (2014). Linking gene expression and membrane lipid composition of *Arabidopsis*. *Plant Cell* 26 915–928. 10.1105/tpc.113.118919 24642935 PMC4001401

[B289] Takos A. M. Jaffe F. W. Jacob S. R. Bogs J. Robinson S. P. Walker A. R. (2006). Light-induced expression of a MYB gene regulates anthocyanin biosynthesis in red apples. *Plant Physiol.* 142 1216–1232. 10.1104/pp.106.088104 17012405 PMC1630764

[B290] Tan H. Zhao Y. (2017). Enrichment of β-carotene from palm oil using supercritical carbon dioxide pretreatment-solvent extraction technique. *LWT Food Sci. Technol.* 83 262–266. 10.1016/j.lwt.2017.05.026

[B291] Taub D. R. Wang X. (2008). Why are nitrogen concentrations in plant tissues lower under elevated CO2 A critical examination of the hypotheses. *J. Integr. Plant Biol.* 50 1365–1374. 10.1111/j.1744-7909.2008.00754.x 19017124

[B292] Tenenboim H. Brotman Y. (2016). Omic relief for the biotically stressed: metabolomics of plant biotic interactions. *Trends Plant Sci.* 21 781–791. 10.1016/j.tplants.2016.04.009 27185334

[B293] Tenenboim H. Burgos A. Willmitzer L. Brotman Y. (2016). Using lipidomics for expanding the knowledge on lipid metabolism in plants. *Biochimie* 130 91–96. 10.1016/j.biochi.2016.06.004 27292697

[B294] Teramura A. H. Sullivan J. H. Ziska L. H. (1990). Interaction of elevated ultraviolet-B radiation and CO2 on productivity and photosynthetic characteristics in wheat, rice, and soybean. *Plant Physiol.* 94 470–475. 10.1104/pp.94.2.470 16667735 PMC1077255

[B295] Thompson A. J. Andrews J. Mulholland B. J. McKee J. M. Hilton H. W. Horridge J. S. (2007). Overproduction of abscisic acid in tomato increases transpiration efficiency and root hydraulic conductivity and influences leaf expansion. *Plant Physiol.* 143 1905–1917. 10.1104/pp.106.093559 17277097 PMC1851808

[B296] Tivendale N. D. Davies N. W. Molesworth P. P. Davidson S. E. Smith J. A. Lowe E. K. (2010). Reassessing the role of N-hydroxytryptamine in auxin biosynthesis. *Plant Physiol.* 154 1957–1965. 10.1104/pp.110.165803 20974893 PMC2996026

[B297] Tohge T. Fernie A. R. (2009). Web-based resources for mass-spectrometry-based metabolomics: a user’s guide. *Phytochemistry.* 70 450–456. 10.1016/j.phytochem.2009.02.004 19285697

[B298] Tohge T. Fernie A. R. (2012). Co-expression and co-responses: within and beyond transcription. *Front. Plant Sci.* 3:248 10.3389/fpls.2012.00248 PMC3492870 23162560

[B299] Tohge T. Fernie A. R. (2015). Metabolomics-inspired insight into developmental, environmental and genetic aspects of tomato fruit chemical composition and quality. *Plant Cell Physiol.* 56 1681–1696. 10.1093/pcp/pcv093 26228272

[B300] Tohge T. Nishiyama Y. Hirai M. Y. Yano M. Nakajima J. Awazuhara M. (2005). Functional genomics by integrated analysis of metabolome and transcriptome of Arabidopsis plants over-expressing an MYB transcription factor. *Plant J.* 42 218–235. 10.1111/j.1365-313X.2005.02371.x 15807784

[B301] Toubiana D. Semel Y. Tohge T. Beleggia R. Cattivelli L. Rosental L. (2012). Metabolic profiling of a mapping population exposes new insights in the regulation of seed metabolism and seed, fruit, and plant relations. *PLoS Genet* 8:e1002612 10.1371/journal.pgen.1002612 PMC3315483 22479206

[B302] Tripathi P. Rabara R. C. Reese R. N. Miller M. A. Rohila J. S. Subramanian S. (2016). A toolbox of genes, proteins, metabolites and promoters for improving drought tolerance in soybean includes the metabolite coumestrol and stomatal development genes. *BMC Genomics* 17:102 10.1186/s12864-016-2420-0 PMC4746818 26861168

[B303] Tripathi P. Rabara R. C. Shulaev V. Shen Q. J. Rushton P. J. (2015). Understanding water-stress responses in soybean using hydroponics system-a systems biology perspective. *Front. Plant Sci.* 6:1145 10.3389/fpls.2015.01145 PMC4685135 26734044

[B304] Tsuchimoto S. Cartagena J. Khemkladngoen N. Singkaravanit S. Kohinata T. Wada N. (2012). Development of transgenic plants in jatropha with drought tolerance. *Plant Biotechnol.* 29 137–143. 10.5511/plantbiotechnology.12.0406d

[B305] Tsugawa H. Cajka T. Kind T. Ma Y. Higgins B. Ikeda K. (2015). MS-DIAL: data-independent MS/MS deconvolution for comprehensive metabolome analysis. *Nat. Methods* 12 523–526. 10.1038/nmeth.3393 25938372 PMC4449330

[B306] Turner M. F. Heuberger A. L. Kirkwood J. S. Collins C. C. Wolfrum E. J. Broeckling C. D. (2016). Non-targeted metabolomics in diverse *Sorghum* breeding lines indicates primary and secondary metabolite profiles are associated with plant biomass accumulation and photosynthesis. *Front. Plant Sci.* 7:953 10.3389/fpls.2016.00953 PMC4939745 27462319

[B307] Upadhyaya P. Tyagi K. Sarma S. Tamboli V. Sreelakshmi Y. Sharma R. (2017). Natural variation in folate levels among tomato (*Solanum lycopersicum*) accessions. *Food Chem.* 217 610–619. 10.1016/j.foodchem.2016.09.031 27664678

[B308] Uprety D. C. Sen S. Dwivedi N. (2010). Rising atmospheric carbon dioxide on grain quality in crop plants. *Physiol. Mol. Biol. Plants* 16 215–227. 10.1007/s12298-010-0029-3 23572972 PMC3550675

[B309] Vadivel A. K. (2015). Gel-based proteomics in plants: time to move on from the tradition. *Front. Plant Sci.* 6:369. 10.3389/fpls.2015.00369 PMC4470439 26136753

[B310] van Dam N. M. Bouwmeester H. J. (2016). Metabolomics in the rhizosphere: tapping into belowground chemical communication. *Trends Plant Sci.* 21 256–265. 10.1016/j.tplants.2016.01.008 26832948

[B311] Vaughn S. F. Berhow M. A. Terry A. I. Ray K. Walter E. L. Jham G. N. (2009). Wild Brazilian mustard (*Brassica juncea* L.) seed oil methyl esters as biodiesel fuel. *J. Am. Oil Chem. Soc.* 86 917–926. 10.1007/s11746-009-1431-2

[B312] Venkatesh T. V. Chassy A. W. Fiehn O. Flint-Garcia S. Zeng Q. Skogerson K. (2016). Metabolomic assessment of key maize resources: GC-MS and NMR profiling of grain from B73 hybrids of the nested association mapping (NAM) founders and of geographically diverse landraces. *J. Agric. Food Chem.* 64 2162–2172. 10.1021/acs.jafc.5b04901 26923484

[B313] Vidal M. Cusick M. E. Barabasi A. L. (2011). Interactome networks and human disease. *Cell* 144 986–998. 10.1016/j.cell.2011.02.016 21414488 PMC3102045

[B314] Villiers F. Ducruix C. Hugouvieux V. Jarno N. Ezan E. Garin J. (2011). Investigating the plant response to cadmium exposure by proteomic and metabolomic approaches. *Proteomics* 11 1650–1663. 10.1002/pmic.201000645 21462346

[B315] Virdi K. S. Laurie J. D. Xu Y. Z. Yu J. Shao M. R. Sanchez R. (2015). Arabidopsis MSH1 mutation alters the epigenome and produces heritable changes in plant growth. *Nat. Commun.* 6:6386 10.1038/ncomms7386 PMC4351625 25722057

[B316] Vriet C. Russinova E. Reuzeau C. (2012). Boosting crop yields with plant steroids. *Plant Cell* 24 842–857. 10.1105/tpc.111.094912 22438020 PMC3336137

[B317] Walker A. R. Lee E. Bogs J. McDavid D. A. J. Thomas M. R. Robinson S. P. (2007). White grapes arose through the mutation of two similar and adjacent regulatory genes. *Plant J.* 49 772–785. 10.1111/j.1365-313X.2006.02997.x 17316172

[B318] Wang A. Yamakake J. Kudo H. Wakasa Y. Hatsuyama Y. Igarashi M. (2009). Null mutation of the MdACS3 gene, coding for a ripening-specific 1-aminocyclopropane-1-carboxylate synthase, leads to long shelf life in apple fruit. *Plant Physiol.* 151 391–399. 10.1104/pp.109.135822 19587104 PMC2735996

[B319] Wang C. Hu S. Gardner C. Lübberstedt T. (2017). Emerging avenues for utilization of exotic germplasm. *Trends Plant Sci.* 22 624–637. 10.1016/j.tplants.2017.04.002 28476651

[B320] Wang H. Nagegowda D. A. Rawat R. Bouvier-Navé P. Guo D. Bach T. J. (2012). Overexpression of *Brassica juncea* wild-type and mutant HMG-CoA synthase 1 in Arabidopsis up-regulates genes in sterol biosynthesis and enhances sterol production and stress tolerance. *Plant Biotechnol. J.* 10 31–42. 10.1111/j.1467-7652.2011.00631.x 21645203

[B321] Wang J. Sun L. Xie L. He Y. Luo T. Sheng L. (2016). Regulation of cuticle formation during fruit development and ripening in “Newhall” navel orange (*Citrus sinensis* Osbeck) revealed by transcriptomic and metabolomic profiling. *Plant Sci.* 243 131–144. 10.1016/j.plantsci.2015.12.010 26795158

[B322] Wang W. Yang X. Tangchaiburana S. Ndeh R. Markham J. E. Tsegaye Y. (2008). An inositolphosphorylceramide synthase is involved in regulation of plant programmed cell death associated with defense in *Arabidopsis*. *Plant Cell* 20 3163–3179. 10.1105/tpc.108.060053 19001565 PMC2613663

[B323] Ward C. Courtney D. (2013). Kiwifruit. Taking its place in the global fruit bowl. *Adv. Food Nutr. Res.* 68 1–14. 10.1016/B978-0-12-394294-4.00001-8 23394979

[B324] Weckwerth W. (2008). Integration of metabolomics and proteomics in molecular plant physiology – coping with the complexity by data-dimensionality reduction. *Physiol. Plant.* 132 176–189. 10.1111/j.1399-3054.2007.01011.x 18251859

[B325] Welti R. Li W. Li M. Sang Y. Biesiada H. Zhou H. E. (2002). Profiling membrane lipids in plant stress responses. *J. Biol. Chem.* 277 31994–32002. 10.1074/jbc.M205375200 12077151

[B326] Weltzin J. F. Bridgham S. D. Pastor J. Chen J. Harth C. (2003). Potential effects of warming and drying on peatland plant community composition. *Glob. Chang. Biol.* 9 141–151. 10.1046/j.1365-2486.2003.00571.x

[B327] Wen W. Li D. Li X. Gao Y. Li W. Li H. (2014). Metabolome-based genome-wide association study of maize kernel leads to novel biochemical insights. *Nat. Commun.* 5:3438 10.1038/ncomms4438 PMC3959190 24633423

[B328] Wen W. Li K. Alseekh S. Omranian N. Zhao L. Zhou Y. (2015). Genetic determinants of the network of primary metabolism and their relationships to plant performance in a maize recombinant inbred line population. *Plant Cell* 27 1839–1856. 10.1105/tpc.15.00208 26187921 PMC4531352

[B329] Wen W. Liu H. Zhou Y. Jin M. Yang N. Li D. (2016). Combining quantitative genetics approaches with regulatory network analysis to dissect the complex metabolism of the Maize Kernel. *Plant Physiol.* 170 136–146. 10.1104/pp.15.01444 26556794 PMC4704590

[B330] Wienkoop S. Morgenthal K. Wolschin F. Scholz M. Selbig J. Weckwerth W. (2008). Integration of metabolomic and proteomic phenotypes: analysis of data covariance dissects starch and RFO metabolism from low and high temperature compensation response in *Arabidopsis thaliana*. *Mol. Cell. Proteomics* 7 1725–1736. 10.1074/mcp.M700273-MCP200 18445580 PMC2556022

[B331] Wilkinson S. Davies W. J. (2002). ABA-based chemical signalling: the co-ordination of responses to stress in plants. *Plant Cell Environ.* 25 195–210. 10.1046/j.0016-8025.2001.00824.x 11841663

[B332] Willis J. D. Mazarei M. Stewart C. N. (2016). Transgenic plant-produced hydrolytic enzymes and the potential of insect gut-derived hydrolases for biofuels. *Front. Plant Sci.* 7:675 10.3389/fpls.2016.00675 PMC4885837 27303411

[B333] Winning H. Roldan-Martłn E. Dragsted L. O. Viereck N. Poulsen M. Sanchez-Moreno C. (2009). An exploratory NMR nutri-metabonomic investigation reveals dimethyl sulfone as a dietary biomarker for onion intake. *Analyst* 134 2344–2351. 10.1039/b918259d 19838425

[B334] Wu C. Y. Trieu A. Radhakrishnan P. Kwok S. F. Harris S. Zhang K. (2008). Brassinosteroids regulate grain filling in rice. *Plant Cell* 20 2130–2145. 10.1105/tpc.107.055087 18708477 PMC2553602

[B335] Wu L. Birch R. G. (2007). Doubled sugar content in sugarcane plants modified to produce a sucrose isomer. *Plant Biotechnol. J.* 5 109–117. 10.1111/j.1467-7652.2006.00224.x 17207261

[B336] Wuolikainen A. Jonsson P. Ahnlund M. Antti H. Marklund S. L. Moritz T. (2016). Multi-platform mass spectrometry analysis of the CSF and plasma metabolomes of rigorously matched amyotrophic lateral sclerosis, Parkinson’s disease and control subjects. *Mol. BioSyst.* 12 1287–1298. 10.1039/c5mb00711a 26883206

[B337] Xavier A. Hall B. Hearst A. A. Cherkauer K. A. Rainey K. M. (2017). Genetic architecture of phenomic-enabled canopy coverage in *Glycine max*. *Genetics* 206 1081–1089. 10.1534/genetics.116.198713 28363978 PMC5499164

[B338] Xianwei S. Zhang X. Sun J. Cao X. (2015). Epigenetic mutation of RAV6 affects leaf angle and seed size in rice. *Plant Physiol.* 169 2118–2128. 10.1104/pp.15.00836 26351308 PMC4634063

[B339] Xie L. J. Chen Q. F. Chen M. X. Yu L. J. Huang L. Chen L. (2015). Unsaturation of very-long-chain ceramides protects plant from hypoxia-induced damages by modulating ethylene signaling in *Arabidopsis*. *PLoS Genet.* 11:e1005143 10.1371/journal.pgen.1005143 PMC4379176 25822663

[B340] Xiong L. Lee M. W. Qi M. Yang Y. (2001). Identification of defense-related rice genes by suppression subtractive hybridization and differential screening. *Mol. Plant Microbe Interact.* 14 685–692. 10.1094/MPMI.2001.14.5.685 11332734

[B341] Yadav S. K. Vanaja M. Reddy P. R. Jyothilakshmi N. Maheswari M. Sharma K. L. (2011). Effect of elevated CO2 levels on some growth parameters and seed quality of groundnut (*Arachis hypogaea* L.). *Indian J. Agric. Biochem.* 24 158–160.

[B342] Yang S. Lu S. H. Yuan Y. J. (2008). Lipidomic analysis reveals differential defense responses of *Taxus cuspidata* cells to two elicitors, methyl jasmonate and cerium (Ce4+). *Biochim. Biophys. Acta* 1781 123–134. 10.1016/j.bbalip.2007.11.005 18179778

[B343] Ye X. Al-Babili S. Klöti A. Zhang J. Lucca P. Beyer P. (2000). Engineering the provitamin A (beta-carotene) biosynthetic pathway into (carotenoid-free) rice endosperm. *Science* 287 303–305. 10.1126/science.287.5451.303 10634784

[B344] Yonekura-Sakakibara K. Saito K. (2006). Review: genetically modified plants for the promotion of human health. *Biotechnol. Lett.* 28 1983–1991. 10.1007/s10529-006-9194-4 17080241

[B345] Yonekura-Sakakibara K. Tohge T. Niida R. Saito K. (2007). Identification of a flavonol 7-O-rhamnosyltransferase gene determining flavonoid pattern in Arabidopsis by transcriptome coexpression analysis and reverse genetics. *J. Biol. Chem.* 282 14932–14941. 10.1074/jbc.M611498200 17314094

[B346] Yun Z. Gao H. Liu P. Liu S. Luo T. Jin S. (2013). Comparative proteomic and metabolomic profiling of citrus fruit with enhancement of disease resistance by post-harvest heat treatment. *BMC Plant Biol.* 13:44 10.1186/1471-2229-13-44 PMC3668225 23497220

[B347] Zalewski W. Galuszka P. Gasparis S. Orczyk W. Nadolska-Orczyk A. (2010). Silencing of the HvCKX1 gene decreases the cytokinin oxidase/dehydrogenase level in barley and leads to higher plant productivity. *J. Exp. Bot.* 61 1839–1851. 10.1093/jxb/erq052 20335409

[B348] Zawirska-Wojtasiak R. Gośliñski M. Szwacka M. Gajc-Wolska J. Mildner-Szkudlarz S. (2009). Aroma evaluation of transgenic, thaumatin II-producing cucumber fruits. *J. Food Sci.* 74 C204–C210. 10.1111/j.1750-3841.2009.01082.x 19397704

[B349] Zhang J. Luo W. Zhao Y. Xu Y. Song S. Chong K. (2016). Comparative metabolomic analysis reveals a reactive oxygen species-dominated dynamic model underlying chilling environment adaptation and tolerance in rice. *New Phytol.* 211 1295–1310. 10.1111/nph.14011 27198693

[B350] Zhang J. Wang X. Yu O. Tang J. Gu X. Wan X. (2011). Metabolic profiling of strawberry (*Fragaria* x *ananassa* Duch.) during fruit development and maturation. *J. Exp. Bot.* 62 1103–1118. 10.1093/jxb/erq343 21041374

[B351] Zhang N. Venkateshwaran M. Boersma M. Harms A. Howes-Podoll M. Den Os D. (2012). Metabolomic profiling reveals suppression of oxylipin biosynthesis during the early stages of legume-rhizobia symbiosis. *FEBS Lett.* 586 3150–3158. 10.1016/j.febslet.2012.06.046 22796495

[B352] Zhang W. Chang J. Lei Z. Huhman D. Sumner L. W. Zhao P. X. (2014). MET-COFEA: a liquid chromatography/mass spectrometry data processing platform for metabolite compound feature extraction and annotation. *Anal*. *Chem.* 86 6245–6253. 10.1021/ac501162k 24856452

[B353] Zhang W. Lei Z. Huhman D. Sumner L. W. Zhao P. X. (2015). MET-XAlign: a metabolite cross-alignment tool for LC/MS-based comparative metabolomics. *Anal. Chem.* 87 9114–9119. 10.1021/acs.analchem.5b01324 26247233

[B354] Zhang X. D. Wang R. P. Zhang F. J. Tao F. Q. Li W. Q. (2013a). Lipid profiling and tolerance to low-temperature stress in *Thellungiella salsuginea* in comparison with *Arabidopsis thaliana*. *Biol. Plant.* 57 149–153. 10.1007/s10535-012-0137-8

[B355] Zhang Y. Butelli E. De Stefano R. Schoonbeek H. J. Magusin A. Pagliarani C. (2013b). Anthocyanins double the shelf life of tomatoes by delaying overripening and reducing susceptibility to gray mold. *Curr. Biol.* 23 1094–1100. 10.1016/j.cub.2013.04.072 23707429 PMC3688073

[B356] Zhong S. Fei Z. Chen Y. R. Zheng Y. Huang M. Vrebalov J. (2013). Single-base resolution methylomes of tomato fruit development reveal epigenome modifications associated with ripening. *Nat. Biotechnol.* 31 154–159. 10.1038/nbt.2462 23354102

[B357] Ziska L. H. Bunce J. A. (2007). Predicting the impact of changing CO2 on crop yields: some thoughts on food. *New Phytol.* 175 607–618. 10.1111/j.1469-8137.2007.02180.x 17688578

[B358] Zivy M. Wienkoop S. Renaut J. Pinheiro C. Goulas E. Carpentier S. (2015). The quest for tolerant varieties: the importance of integrating “omics” techniques to phenotyping. *Front. Plant Sci.* 6:448 10.3389/fpls.2015.00448 PMC4496562 26217344

[B359] Zoeller M. Stingl N. Krischke M. Fekete A. Waller F. Berger S. (2012). Lipid profiling of the Arabidopsis hypersensitive response reveals specific lipid peroxidation and fragmentation processes: biogenesis of pimelic and azelaic acid. *Plant Physiol.* 160 365–378. 10.1104/pp.112.202846 22822212 PMC3440211

[B360] Zrodnikov Y. Davis C. E. (2012). The highs and lows of FAIMS: predictions and future trends for high field asymmetric waveform ion mobility spectrometry. *J. Nanomed. Nanotechnol.* 3:109e 10.4172/2157-7439.1000e109 PMC3807102 24163785

